# Predictive estimation of economic outcome changes associated with legal frameworks and public health policy conditions

**DOI:** 10.3389/fpubh.2026.1781611

**Published:** 2026-07-23

**Authors:** Peiran Lu, Songling Liu, Pai Pang

**Affiliations:** 1 Law School, Beijing Foreign Studies University, Beijing, China; 2 Institute of Automation, Chinese Academy of Sciences, Beijing, China

**Keywords:** agent driven policy simulation, Counterfactual Policy Optimizer, economic and legal frameworks, public health policy analysis, uncertainty aware policy-associated outcome prediction

## Abstract

**Introduction:**

Public health policies are implemented within legal and regulatory environments associated with heterogeneous economic and health related outcomes across regions and time. Existing policy evaluation approaches often have limited capacity to represent regional heterogeneity, temporal policy patterns, and uncertainty in observed policy associated outcome changes. To address this issue, this study proposes a predictive computational framework for estimating subsequent economic outcome changes under observed public health policy, legal, and regulatory conditions.

**Methods:**

The empirical task is formulated as supervised temporal regression using region time observations constructed from the Oxford COVID-19 Government Response Tracker and COVID-19 US State Policy datasets. The proposed Counterfactual Policy Optimizer (CPO) represents legal frameworks as policy constraints and regulatory variables, and combines manifold constraint regularization, agent driven policy interaction modeling, and probabilistic outcome forecasting. In this study, the term counterfactual refers to model based scenario comparison within the observed data distribution and feasible policy space, and does not imply causal identification in the econometric or structural causal sense.

**Results and discussion:**

The training objective integrates prediction accuracy, uncertainty modeling, utility based policy comparison, and constraint regularization. Experimental results indicate that the proposed framework achieves lower prediction error and stronger trend consistency than traditional econometric, machine learning, and temporal deep learning baselines under the same observational prediction setting. Additional policy related indicators indicate that legal feasibility, implementation conditions, and uncertainty aware forecasting can support structured predictive evaluation of public health policy associated economic outcome changes. The study provides a reproducible framework for examining temporal associations between legally structured public health policy conditions and subsequent economic outcome changes.

## 1 Introduction

The observed economic outcome1 changes associated with legal frameworks and public health policy conditions have garnered significant attention in recent years due to their profound implications for societal wellbeing and resource allocation (1). Public health policies are critical for addressing health crises and are often implemented in ways that coincide with changes in economic stability, resource allocation, and social development (2). Legal frameworks, as the institutional backbone of these policies, define their enforceability, equity, administrative scope, and implementation boundaries (3). However, the relationships among legal structures, public health interventions, and economic outcomes remain complex, heterogeneous, and difficult to characterize across regions and time. These frameworks are associated with direct costs and benefits of health interventions, and they may also correspond to longer-term economic resilience through workforce productivity, healthcare expenditures, and social equity (4). Furthermore, the global nature of public health challenges, such as pandemics, underscores the need for robust legal mechanisms that can adapt to diverse economic and cultural contexts (5). Therefore, examining the predictive associations between legal frameworks, public health policy conditions, and subsequent economic outcome changes is essential for supporting policy analysis that is sustainable, equitable, and empirically transparent (6). Legal frameworks shape public health policy implementation through several institutional mechanisms. Laws and regulations define the authority of public agencies, allocate administrative responsibilities across levels of government, establish compliance obligations for individuals and organizations, specify enforcement procedures, and delimit the scope of permissible public action. They also provide safeguards for vulnerable populations by setting requirements related to access, non-discrimination, proportionality, accountability, and procedural legitimacy. These legal mechanisms influence not only whether a public health intervention can be formally adopted, but also whether it can be implemented consistently and equitably across regions. Therefore, representing legal and regulatory conditions as constraints and policy variables is important for analyzing public health measures in a manner that reflects institutional feasibility rather than abstract policy design alone.

In the initial phase of research, efforts were concentrated on developing structured models to evaluate economic outcome changes observed under different legal and public health policy conditions (7). These models utilized predefined rules and expert insights to simulate interactions among legal structures, economic factors, and health outcomes. By incorporating domain-specific knowledge, researchers could assess possible relationships between legal interventions, public health indicators, and economic metrics (8). However, these models were often limited by their static nature, which restricted their ability to adapt to the evolving and complex landscape of real-world public health challenges (9). The manual construction and maintenance of these models also posed significant challenges, making them resource-intensive and susceptible to inaccuracies (10). As the need for more adaptable approaches became apparent, researchers turned to advanced computational techniques to study economic outcome changes associated with legal frameworks and public health policy conditions (11). By leveraging large datasets, these methods aimed to identify temporal patterns and statistical relationships that were not easily discernible through traditional models (12). Statistical models and supervised learning techniques were employed to enhance the predictive accuracy and granularity of economic outcome estimation under observed legal and policy conditions (13). For instance, these approaches have been instrumental in supporting the predictive evaluation of public health policies by integrating diverse data sources, such as demographic information and economic indicators (1). Despite their advantages, challenges related to data quality, interpretability, temporal generalizability, and uncertainty remained, necessitating further refinement of these methodologies (2).

The advent of deep learning and pre-trained models has further transformed the computational analysis of legal frameworks, public health policy conditions, and their associated economic outcome changes (3). These advanced methods utilize neural networks to automatically extract features and learn complex representations from extensive data sources, including legal texts, policy records, and economic reports. Pre-trained models, such as transformers, have facilitated transfer learning, allowing models to be fine-tuned for specific prediction tasks with greater precision and scalability (4). This has enabled researchers to explore intricate statistical interactions between legal, economic, and health variables with improved predictive capacity (5). However, the high computational demands and the necessity for substantial labeled data remain significant barriers to widespread adoption (6). The opaque nature of deep learning models also presents challenges regarding accountability, interpretability, and responsible use, especially within critical policy-making contexts (7).

Based on the limitations discussed above, this study proposes a computational policy analysis framework for estimating subsequent economic outcome changes under observed public health policy, legal, and regulatory conditions. The core research objective is to examine how legally structured public health interventions are predictively associated with subsequent economic outcome changes across regions and time. Legal frameworks are represented as policy constraints and regulatory variables, public health policy provides the substantive application context, and economic response is treated as the primary outcome to be estimated. In this setting, machine learning and deep learning techniques are used as modeling tools for capturing non-linear temporal relationships, predictive uncertainty, and model-based policy scenario responses, rather than as the independent disciplinary focus of the study. The proposed framework integrates structured legal and policy knowledge with data-driven temporal prediction. This design enables the model to account for legally feasible policy spaces, regional heterogeneity, and dynamic economic responses observed after public health interventions. By incorporating constraint regularization, agent-driven policy interaction modeling, and probabilistic outcome forecasting, the framework aims to provide a more coherent analytical tool for policy-associated outcome prediction. Because the empirical analysis is based on observational panel data and supervised temporal regression, the proposed framework is not intended to identify causal effects in the econometric or structural causal sense. Instead, it estimates predictive associations between observed policy, legal, regulatory, economic, and public health variables and subsequent economic outcome changes. The term counterfactual in this study refers to model-based scenario comparison within the observed data distribution and feasible legal-policy space, rather than to causally identified potential outcomes. The goal is to support evidence-based predictive evaluation of public health policies under complex legal and economic conditions, while maintaining consistency between the research question, task definition, datasets, and evaluation metrics. Economic outcome change is selected as the primary prediction target because it provides an observable and policy relevant signal of intervention burden, regional resilience, and socioeconomic sustainability. This choice does not imply that economic considerations override health objectives. Rather, public health policies often depend on sustained resources, administrative capacity, and social cooperation. Economic deterioration may weaken health system financing, reduce household access to care, and lower compliance with public measures, while economically sustainable interventions may be easier to maintain during prolonged public health emergencies. Accordingly, the economic prediction target is used to examine how public health policy conditions are associated with subsequent socioeconomic responses that may affect the durability and implementation quality of health protection measures.

The public health relevance of predicting subsequent economic outcome changes lies in the close connection between economic conditions and the practical sustainability of health interventions. Economic responses following public health measures are not merely contextual factors outside the health domain. They may influence the fiscal resources available for prevention programs, the operational capacity of health systems, the accessibility of services for affected populations, and the willingness of communities to comply with public measures. For example, substantial adverse economic responses may reduce the resources available for testing, vaccination, treatment, workforce protection, and emergency preparedness. They may also increase barriers to care for socially and economically vulnerable groups, thereby weakening the equity and effectiveness of public health interventions. In this sense, estimating economic outcome changes can support public health governance by identifying policy conditions under which health protection, institutional feasibility, resource capacity, and social acceptance are more likely to remain compatible over time.

The main contributions are summarized as follows:

- A predictive public health policy outcome estimation framework is proposed to analyze how legal and regulatory conditions are associated with subsequent economic outcome changes after policy interventions.
- The proposed Counterfactual Policy Optimizer (CPO) combines constraint-based policy representation, agent-driven interaction modeling, and probabilistic outcome forecasting to capture dynamic policy-associated response patterns.
- The empirical task is clearly defined as supervised temporal regression, where policy variables and regional characteristics are used to predict continuous economic outcome changes without making causal identification claims.
- Experiments on OxCGRT and CUSP demonstrate that the proposed method improves predictive accuracy and trend consistency under a unified observational policy outcome estimation setting.

**Figure 1:**
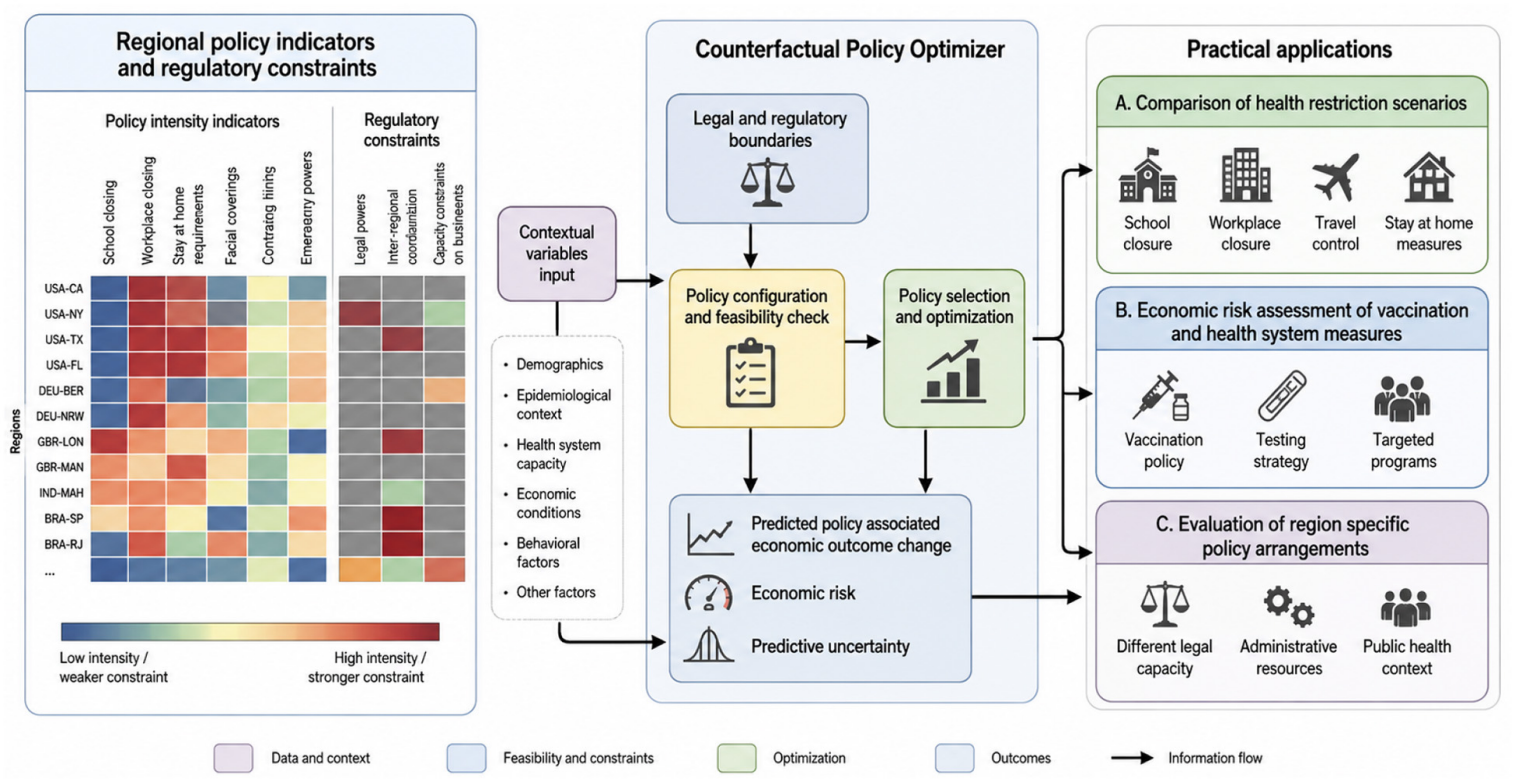
Illustrative applications of the Counterfactual Policy Optimizer for public health policy decision support. The figure summarizes how regional policy indicators, regulatory constraints, and contextual variables are integrated into the CPO workflow. The model checks policy configurations against legal and regulatory boundaries, performs constrained policy selection, and generates predicted policy associated economic outcome change, economic risk, and predictive uncertainty. These outputs can support comparison of health restriction scenarios, economic risk assessment of vaccination and health system measures, and evaluation of region specific policy arrangements.

The practical use of the proposed Counterfactual Policy Optimizer is further illustrated in Figure 1. The figure shows how regional policy indicators, regulatory constraints, and contextual variables can be organized as inputs for feasibility aware policy analysis. Policy intensity indicators, such as school closure, workplace closure, stay at home requirements, facial covering rules, contact tracing, gathering limits, and emergency powers, are considered together with legal powers, inter regional coordination, and capacity constraints. These variables enter the Counterfactual Policy Optimizer, where legal and regulatory boundaries first define the feasible policy space, policy configurations are then checked for institutional feasibility, and policy selection is performed within the constrained decision space. The model subsequently estimates predicted policy associated economic outcome change, economic risk, and predictive uncertainty. These outputs can support three practical decision settings: comparison of health restriction scenarios, economic risk assessment of vaccination and health system measures, and evaluation of region specific policy arrangements. This illustration clarifies that the proposed framework is intended to provide feasibility aware predictive decision support, rather than causal estimation of policy effects.

## 2 Related work

### 2.1 Legal frameworks and health equity

**Figure 2:**
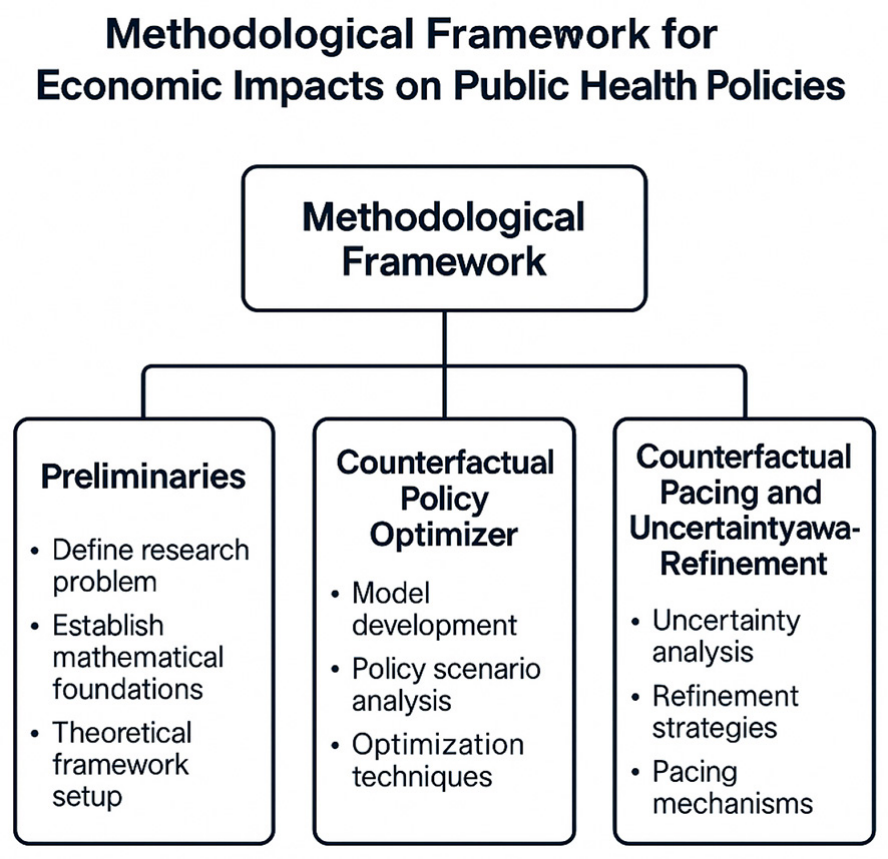
The diagram illustrates the methodological framework for assessing the economic outcome changes of legal frameworks on public health policies. It is structured into three main components: Preliminaries, Counterfactual Policy Optimizer, and Counterfactual Pacing and Probabilistic Policy Outcome Refinement. Each component addresses specific aspects of the research, from defining the research problem and establishing mathematical foundations to developing models and refining strategies for policy analysis.

The intersection of legal frameworks and health equity represents a critical area of study in understanding the economic outcome changes of public health policy (Figure 2). Legal frameworks, including regulations, statutes, and judicial decisions, play a pivotal role in shaping the distribution of health resources and access to care (8). Health equity, defined as the absence of avoidable or remediable differences among groups of people, is often influenced by the legal structures that govern healthcare systems, insurance coverage, and public health interventions (9). Economic disparities, often exacerbated by inequitable legal frameworks, can lead to significant variations in health outcomes across different populations (10). One of the primary mechanisms through which legal frameworks influence health equity is the regulation of healthcare financing and insurance systems (11). Laws that mandate universal health coverage or expand Medicaid eligibility have been shown to reduce economic barriers to accessing care, thereby improving health outcomes for low-income populations (12). Conversely, restrictive legal policies that limit access to affordable healthcare can perpetuate economic inequalities and exacerbate health disparities (13). The economic burden of untreated illnesses, particularly among marginalized groups, often results in higher long-term costs for both individuals and healthcare systems (14). Another critical aspect is the role of anti-discrimination laws in promoting health equity (15). Legal protections against discrimination based on race, gender, disability, or socioeconomic status can mitigate the economic and social barriers that hinder access to healthcare services (16). The enforcement of the Americans with Disabilities Act (ADA) in the United States has improved access to healthcare facilities and services for individuals with disabilities, reducing economic disparities in health outcomes. However, the effectiveness of such legal protections often depends on robust enforcement mechanisms and adequate funding, highlighting the economic implications of legal frameworks on public health policy. The economic outcome changes of legal frameworks on health equity also extend to the regulation of social determinants of health, such as housing, education, and employment (17). Laws that address these determinants, such as minimum wage legislation or affordable housing policies, can have significant downstream effects on public health (18). Research has shown that increasing the minimum wage is associated with improved health outcomes, as it reduces economic stress and enables individuals to afford healthier lifestyles (19). However, the implementation of such policies often involves trade-offs, including potential economic costs for businesses and governments, which must be carefully considered in the context of public health policy (20).

### 2.2 Economic costs of regulatory compliance

**Figure 3:**
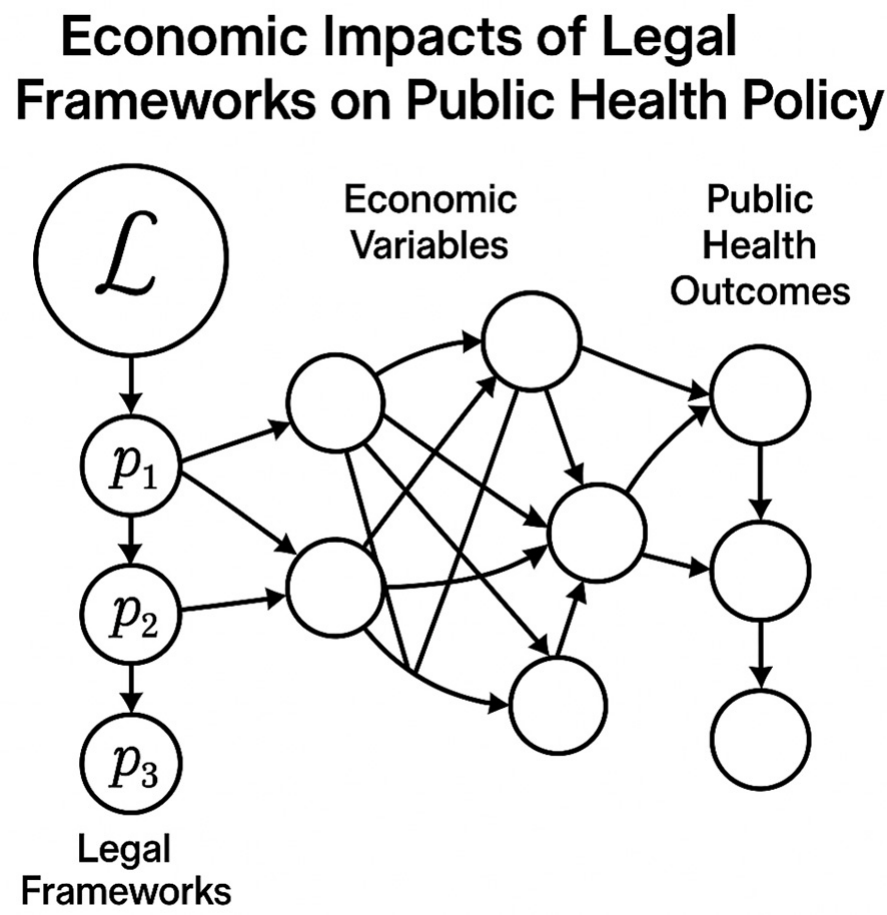
The diagram illustrates the dynamic interactions between legal frameworks, economic variables, and public health outcomes. Legal frameworks, characterized by regulatory parameters, influence economic indicators, which in turn affect public health metrics. This interconnected system forms the basis for evaluating the effectiveness of policy interventions using the Counterfactual Policy Optimizer.

The economic costs associated with regulatory compliance represent a significant factor in evaluating the effectiveness of public health policies (21) (Figure 3). Legal frameworks often impose specific requirements on healthcare providers, businesses, and public institutions to ensure the implementation of health policies (22). These requirements, while essential for achieving public health objectives, can entail substantial economic costs, including administrative expenses, infrastructure investments, and workforce training. Understanding these costs is crucial for assessing the feasibility and sustainability of public health interventions. One of the primary areas where regulatory compliance incurs economic costs is in the healthcare sector. Laws mandating electronic health record (EHR) systems have improved the efficiency and quality of care but have also imposed significant financial burdens on healthcare providers (23). The initial costs of implementing EHR systems, including software acquisition, staff training, and system maintenance, can be prohibitive for smaller healthcare facilities (8). These economic challenges can hinder the adoption of such technologies, thereby limiting the effectiveness of public health policies aimed at improving healthcare delivery (9). Another area of concern is the economic impact of compliance with environmental health regulations (10). Legal frameworks that address environmental determinants of health, such as air and water quality standards, often require businesses and industries to invest in pollution control technologies and sustainable practices (11). While these regulations are essential for protecting public health, the associated costs can be substantial, particularly for small and medium-sized enterprises (12). The economic burden of compliance may also lead to unintended consequences, such as job losses or increased consumer prices, which can undermine the broader goals of public health policies (13). The economic costs of regulatory compliance are also evident in the implementation of workplace health and safety laws (14). Regulations that mandate safety training, protective equipment, and workplace inspections are critical for preventing occupational injuries and illnesses (15). However, the financial implications of these requirements can be significant, particularly for industries with high-risk work environments (16). Balancing the economic costs of compliance with the benefits of improved worker health and productivity is a key challenge for policymakers. Moreover, the effectiveness of such regulations often depends on the availability of resources for enforcement and monitoring, which further underscores the economic dimensions of regulatory compliance.

### 2.3 Legal incentives and public health outcomes

**Figure 4:**
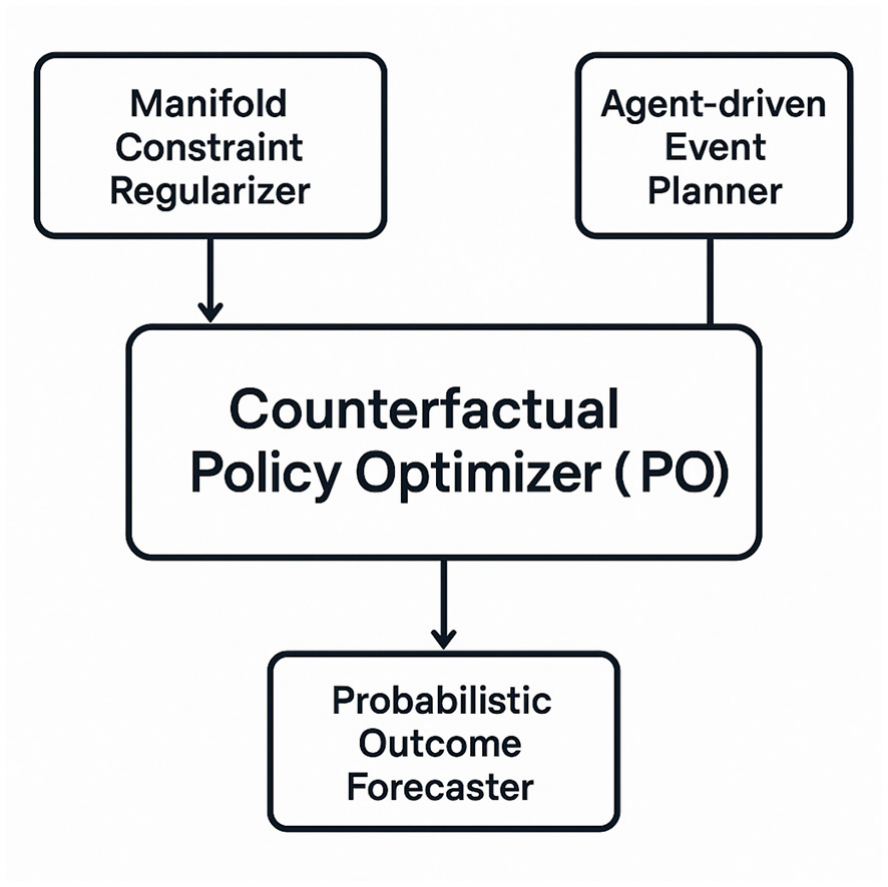
The Counterfactual Policy Optimizer (CPO) framework integrates three core modules: the Manifold Constraint Regularizer, the Agent-driven Event Planner, and the Probabilistic Outcome Forecaster. These modules collectively enable the simulation of counterfactual scenarios, refinement of policy strategies, and prediction of outcomes under varying legal and economic conditions. The CPO ensures that policy evaluations and optimizations remain within realistic and legally permissible boundaries.

The use of legal incentives to influence public health outcomes is a prominent area of research in understanding the economic outcome changes of legal frameworks (17) (Figure 4). Incentives, which can take the form of tax benefits, subsidies, or penalties, are designed to encourage behaviors that promote public health while discouraging practices that pose health risks (18). The economic implications of these incentives are multifaceted, affecting individuals, businesses, and governments in various ways (19). One of the most widely studied examples of legal incentives is the use of taxation to reduce the consumption of harmful products, such as tobacco, alcohol, and sugary beverages (20). Excise taxes on these products have been shown to decrease consumption rates, leading to improved public health outcomes and reduced healthcare costs (21). However, the economic impact of such taxes is not uniformly distributed, as they often disproportionately affect low-income populations (22). This raises important questions about the equity and fairness of using taxation as a public health tool, as well as the potential for regressive economic effects. Subsidies and financial incentives for healthy behaviors represent another critical aspect of legal frameworks. Laws that provide tax credits for employers offering workplace wellness programs or subsidies for individuals purchasing gym memberships can promote healthier lifestyles and reduce the economic burden of chronic diseases. These incentives not only improve public health outcomes but also generate economic benefits by reducing healthcare expenditures and increasing workforce productivity (23). However, the effectiveness of such incentives often depends on their design and implementation, as well as the availability of resources to support their adoption (8). Penalties for non-compliance with public health regulations also play a significant role in shaping health outcomes (9). Fines for failing to meet vaccination requirements or penalties for violating food safety standards are designed to ensure adherence to public health policies (10). While these penalties can be effective in promoting compliance, they also have economic implications for individuals and businesses (11). The financial burden of penalties may disproportionately affect small businesses or low-income individuals, highlighting the need for equitable and economically sustainable approaches to enforcement (12). The economic outcome changes of legal incentives on public health outcomes are further influenced by the broader policy context, including the availability of complementary measures such as education and outreach programs (13). The effectiveness of tobacco taxes in reducing smoking rates is often enhanced by public awareness campaigns and smoking cessation programs (14). This underscores the importance of integrating legal incentives with other public health strategies to achieve optimal outcomes while minimizing economic disparities (15).

### 2.4 Predictive analytics and digital governance

**Figure 5:**
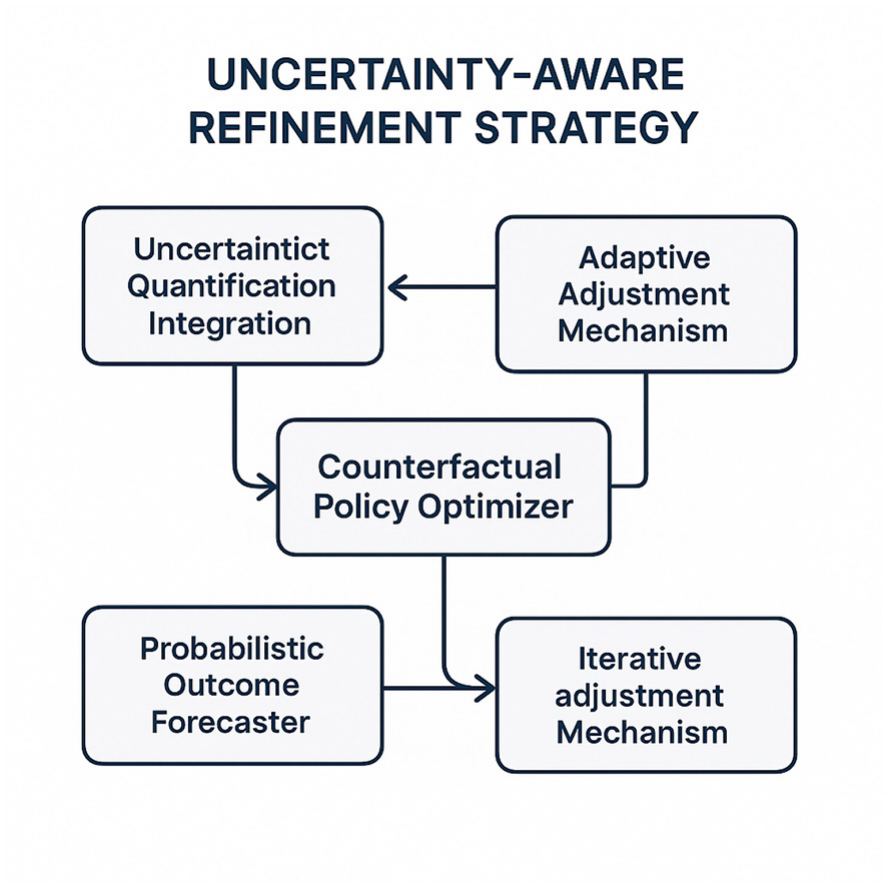
The diagram illustrates the components and workflow of the Probabilistic Policy Outcome Refinement strategy within the Counterfactual Policy Optimizer. Key modules include Uncertainty Quantification Integration, Probabilistic Outcome Forecaster, Adaptive Adjustment Mechanism, and Iterative Adjustment Mechanism. These modules interact to refine policy predictions and recommendations by incorporating uncertainty quantification and adaptive adjustments, ensuring robust outcomes in complex policy environments.

Recent research has shown that algorithmic systems can support public sector decision making by assisting administrative analysis, policy monitoring, and the processing of complex public data (24) (Figure 5). Artificial intelligence has also been discussed as a governance tool that operates across multiple levels of public administration, including institutional actors, organizational processes, and broader policy environments (25). More recent studies further note that the integration of artificial intelligence into public administration may improve analytical capacity, but it also introduces vulnerabilities related to implementation quality, institutional preparedness, and governance risk (26). Research on government decision making highlights the opportunities of artificial intelligence for service delivery, forecasting, and decision support, while also emphasizing unresolved challenges for public organizations (27). These developments are relevant to public health policy analysis because public health emergencies often require timely decisions based on heterogeneous regional information, incomplete evidence, and limited administrative resources. Predictive models may help identify temporal patterns in policy responses, compare possible outcome trajectories, and support the allocation of public resources under uncertainty. The literature also emphasizes that algorithmic decision support in the public sector raises concerns related to transparency, accountability, institutional oversight, bias, and public legitimacy (28). These concerns are particularly important in public health, where policy recommendations may affect access to essential services, the distribution of health resources, and the legitimacy of emergency interventions. Many existing predictive policy models mainly focus on statistical accuracy, operational efficiency, or administrative automation. However, legal feasibility, administrative enforceability, equity protection, and institutional constraints are often treated as external governance issues rather than as internal components of the modeling structure. The proposed Counterfactual Policy Optimizer differs from general algorithmic policy models in three main respects. It embeds prediction within a legally constrained policy space through the Manifold Constraint Regularizer, rather than treating policy variables as unconstrained numerical features. It models policy sensitive interactions among public health interventions, regional conditions, and institutional contexts through the Agent Driven Event Planner. It incorporates uncertainty aware forecasting through the Probabilistic Outcome Forecaster, so that policy associated economic outcome changes are estimated together with predictive uncertainty. The proposed framework is positioned as a legally constrained and uncertainty aware predictive framework for public health policy outcome estimation, rather than as a general artificial intelligence tool for public administration.

Recent literature on digital transformation and regional disparities further indicates that the effectiveness of predictive tools depends on whether they can account for territorial heterogeneity, institutional conditions, and the digital readiness of public systems. Regional economic growth models in the digital decade suggest that digital development is uneven across regions and is shaped by economic structure, institutional capacity, infrastructure, and technological preparedness (29). Research on the transition to Industry 5.0 also emphasizes that digitization follows an uneven regional geography, which requires analytical frameworks capable of capturing spatial disparities and region specific development conditions (30). These findings are relevant to public health policy prediction because data quality, administrative capacity, policy implementation resources, and digital infrastructure may differ substantially across jurisdictions. Predictive policy models should not treat all regions as institutionally equivalent, but should incorporate regional context, legal and regulatory conditions, and public system readiness into the estimation process.

### 2.5 Research gaps

Although the existing literature provides important insights into the legal, economic, and public health dimensions of policy evaluation, several limitations remain. Studies in public health law and legal regulation have extensively discussed institutional authority, legal legitimacy, rights protection, and enforcement mechanisms, but they often provide limited formal representation of how legal and regulatory arrangements are associated with dynamic economic outcome changes across regions and time. As a result, the economic responses that follow the implementation of legally structured public health policies are not always modeled in a temporally explicit manner. Econometric studies have made important contributions to public policy evaluation, especially when credible identification strategies are available. However, many such approaches depend on strong assumptions about treatment assignment, comparison groups, parallel trends, or exogenous variation. In complex public health settings, these assumptions may be difficult to satisfy because policies are often adopted in response to rapidly changing epidemiological, institutional, and economic conditions. Moreover, conventional econometric specifications may have limited flexibility in representing non-linear temporal responses, heterogeneous regional trajectories, and uncertainty in observed policy associated outcome changes. Recent machine learning and deep learning approaches have improved the ability to extract complex patterns from large scale policy and regional datasets. Nevertheless, many predictive models treat policy variables as unconstrained numerical inputs and do not explicitly incorporate legal feasibility, administrative enforceability, institutional boundaries, or rights based constraints. This limitation is important in public health policy analysis because a statistically favorable policy scenario may not be legally permissible, administratively feasible, or equitable in practice. These gaps indicate the need for a framework that can jointly represent legal and regulatory constraints, economic response variables, temporal policy dynamics, regional heterogeneity, and predictive uncertainty. The proposed Counterfactual Policy Optimizer is designed to address this need by integrating constraint based policy representation, agent driven policy interaction modeling, and probabilistic outcome forecasting within a unified supervised temporal regression framework.

To further connect the reviewed literature with the proposed methodology, each component of the Counterfactual Policy Optimizer is motivated by a specific strand of prior research. The literature on legal frameworks and health equity supports the introduction of constraint based policy representation. Since public health policies must remain consistent with legal authority, non-discrimination principles, proportionality, accountability, and the protection of vulnerable groups, policy prediction should not be performed in an unconstrained space. This consideration motivates the Manifold Constraint Regularizer, which restricts policy configurations and predicted responses to legally and institutionally feasible regions. The literature on the economic costs of regulatory compliance motivates the inclusion of economic variables as central elements of the prediction task. Public health interventions may generate administrative costs, fiscal burdens, organizational adjustments, and compliance related expenses for governments, institutions, firms, and households. These economic responses can influence the sustainability of interventions, the capacity of health systems, and the willingness of affected actors to comply with policy requirements. The proposed framework treats economic outcome change as the primary prediction target and incorporates regional economic characteristics into the temporal input representation. The literature on legal incentives and public health outcomes motivates the modeling of alternative policy scenarios. Taxes, subsidies, penalties, mandates, and other regulatory instruments may produce different behavioral and economic responses depending on regional conditions and institutional contexts. This observation supports the Agent Driven Event Planner and the Probabilistic Outcome Forecaster, which are used to represent policy sensitive interactions and estimate uncertainty aware outcome changes under alternative feasible policy configurations. In this way, the literature on equity, compliance costs, and legal incentives is directly linked to the constraint design, economic variable selection, and policy scenario modeling of the proposed framework.

## 3 Method

### 3.1 Overview

This section presents the methodological framework for estimating subsequent economic outcome changes associated with public health policies implemented under legal and regulatory constraints. The central research question is how legally structured public health interventions are associated with subsequent economic outcome changes across regions and time. Accordingly, the study is positioned as a public health policy-associated outcome prediction study supported by computational modeling. Legal frameworks define policy constraints and regulatory variables, public health policy provides the substantive application context, and economic response is treated as the primary outcome to be estimated. Machine learning and deep learning techniques are used as analytical tools for modeling non-linear temporal relationships, uncertainty, and counterfactual policy responses, rather than as the independent disciplinary focus of the study. Because the empirical analysis is based on observational panel data and supervised temporal regression, the proposed framework should not be interpreted as identifying causal effects of legal frameworks or public health policies. The model does not rely on a structural causal model, randomized intervention, instrumental variable, regression discontinuity design, synthetic control construction, or other exogenous source of variation required for causal identification. Instead, it estimates predictive associations between historically observed policy, legal, regulatory, economic, and public health variables and subsequent economic outcome changes. The counterfactual scenarios considered in this study are therefore model-based predictive scenarios constrained by the observed data distribution and feasible legal-policy space, rather than causally identified potential outcomes. Accordingly, terms such as policy response, outcome change, and scenario comparison are used in a predictive and associational sense throughout the revised manuscript.

The methodology is organized into three principal components: Preliminaries (Section 3.2), Counterfactual Policy Optimizer (Section 3.3), and Counterfactual Pacing and Probabilistic Policy Outcome Refinement (Section 3.4). These components correspond to the theoretical formulation, computational modeling, and iterative refinement stages of the proposed policy-associated outcome prediction framework. This organization is intended to keep the legal, economic, public health, and computational elements aligned with a single empirical task, namely supervised temporal regression for predicting continuous economic outcome changes after public health policy interventions.

In Preliminaries (Section 3.2), the research problem is formalized by defining the mathematical and theoretical foundations required for policy-associated outcome prediction. Legal and regulatory arrangements are represented through structured policy parameters and feasibility constraints. Economic variables and public health indicators are represented as regional state variables that evolve over time. This formulation provides the basis for modeling how policy interventions, legal constraints, regional conditions, and public health dynamics jointly shape economic responses. By defining the input, output, state transition, utility function, and feasibility manifold, this subsection establishes a coherent problem space for the subsequent model design.

The Counterfactual Policy Optimizer (Section 3.3) provides the core modeling framework for estimating policy-associated outcome changes under alternative legal and regulatory conditions. It contains three modules: the Manifold Constraint Regularizer, the Agent Driven Event Planner, and the Probabilistic Outcome Forecaster. The Manifold Constraint Regularizer restricts predictions and policy configurations to legally and economically feasible regions. The Agent Driven Event Planner models interactions among policy agents, regional conditions, and external events. The Probabilistic Outcome Forecaster predicts economic outcome changes while accounting for uncertainty in observed policy response data. Together, these modules support counterfactual policy-associated outcome prediction within a constrained and interpretable modeling structure.

In Counterfactual Pacing and Probabilistic Policy Outcome Refinement (Section 3.4), the framework further refines policy simulations and prediction results. Counterfactual pacing is used to generate and compare alternative policy scenarios over time, so that the temporal structure of policy implementation and economic response can be represented more explicitly. Probabilistic policy outcome refinement is used to incorporate uncertainty quantification and adaptive adjustment into the prediction process. These mechanisms improve the robustness of the model when policy-associated outcome changes are heterogeneous across regions, time periods, and institutional settings.

The integration of these components provides a structured approach for public health policy-associated outcome prediction under legal and economic constraints. The Preliminaries define the task and feasible decision space, the Counterfactual Policy Optimizer estimates policy sensitive economic responses, and the Counterfactual Pacing and Probabilistic Policy Outcome Refinement improves prediction stability under uncertainty. This structure clarifies that the proposed framework is not intended to replace legal reasoning, economic theory, or public health judgment. Instead, it provides a computational tool for comparing policy consequences within a legally constrained and empirically observable decision space.

A further methodological clarification is necessary regarding the use of a utility based optimization framework in the present study. Public health policy evaluation typically involves the simultaneous consideration of multiple objectives, including the improvement of health outcomes, the mitigation of adverse economic effects, the preservation of institutional feasibility, and the allocation of limited public resources under uncertainty. In such contexts, a utility based framework provides a structured analytical device for representing policy consequences that unfold across different domains and time horizons. Its role is not to reduce public health governance to a single economic logic, but to make explicit the competing considerations that arise when alternative legal and policy arrangements must be compared within a common evaluative structure. The theoretical justification for this approach lies in the nature of the problem addressed in this study. The aim is not to provide a complete moral theory of public health policy, but to develop a formal method for comparative analysis under conditions of legal, economic, and administrative complexity. Within such a setting, optimization serves as a disciplined procedure for identifying policy configurations that perform better within a constrained and predefined space of admissible choices. The utility based formulation therefore functions as a methodological instrument for consequence analysis, counterfactual comparison, and policy learning, while leaving broader questions of legal legitimacy and ethical priority to the normative frameworks that define the boundaries of acceptable policy choice.

### 3.2 Preliminaries

To formalize predictive estimation of economic outcome changes associated with legal and public health policy conditions, we begin by defining the problem within a mathematical framework. This formalization provides the foundation for the subsequent development of the Counterfactual Policy Optimizer, as well as its associated modules and strategies. The problem is inherently complex, involving dynamic interactions between legal interventions, economic variables, and public health outcomes. Below, we define the key components and notations that will be used throughout this work.

Let L represent the set of legal frameworks under consideration, where each framework *l* ∈ L is characterized by a set of regulatory parameters **p**_*l*_ = {*p*_*l*, 1_, *p*_*l*, 2_, …, *p*_*l, m*_}. These parameters may include tax rates, subsidy levels, or enforcement intensities. To operationalize legal frameworks in the empirical model, these regulatory parameters are encoded according to their observable forms in the policy records. Binary variables are used to indicate the presence or absence of a legal or regulatory measure, such as a mask mandate, school closure rule, travel restriction, or vaccination requirement. Ordinal variables are used when the source datasets record intervention intensity, such as no measure, recommendation, partial requirement, or mandatory requirement. Severity and strictness indices are used to summarize the overall restrictiveness of policy packages when multiple legal measures are implemented at the same time. Enforcement intensity variables are used to represent the degree to which a measure is implemented, monitored, or sanctioned when such information is available. Coverage indicators are also used to distinguish whether a measure applies nationally, subnationally, to selected regions, or to specific population or institutional groups. These encoded variables are integrated into the policy parameter vector **p**_*l*_, so that legal frameworks can be used as structured model inputs while retaining their institutional meaning. The economic environment is represented by a set of variables E = {*e*_1_, *e*_2_, …, *e*_*n*_}, where each *e*_*i*_ corresponds to a specific economic indicator, such as GDP, unemployment rate, or healthcare expenditure. Similarly, public health outcomes are denoted by H = {*h*_1_, *h*_2_, …, *h*_*k*_}, where each *h*_*j*_ represents a measurable health metric, such as infection rates, mortality rates, or vaccination coverage.

The relationship between legal frameworks, economic variables, and public health outcomes can be modeled as a dynamic system. Let **x**_*t*_ = (**e**_*t*_, **h**_*t*_) denote the state of the system at time *t*, where ${\text{e}}_{t}\in {\mathbb{R}}^{n}$ and ${\text{h}}_{t}\in {\mathbb{R}}^{k}$ are the economic and health state vectors, respectively. The evolution of the system is governed by a transition function *f*, such that Equation 1:


$$\begin{array}{l} {\text{x}}_{t+1}=f\left({\text{x}}_{t},{\text{p}}_{l},{\text{u}}_{t}\right)+\epsilon_{t}, \end{array}$$ (1)

where **u**_*t*_ represents external interventions, and **ϵ**_*t*_ is a stochastic noise term capturing unmodeled dynamics. This equation means that the economic and public health state observed in the next period is predicted from the current regional state, the legal and regulatory policy parameters, and external interventions. In policy terms, it represents the idea that economic and health conditions evolve over time under the combined influence of existing regional conditions, public health measures, legal implementation settings, and unobserved shocks. The stochastic term is included to acknowledge that not all relevant social, institutional, or economic factors can be fully measured in observational policy data.

The primary objective of this study is to evaluate the effectiveness of a given legal framework *l* ∈ L in optimizing public health outcomes H while minimizing adverse economic outcome changes E. To achieve this, we define a utility function *U*(**e**, **h**) that quantifies the trade-off between economic and health objectives (Equation 2):


$$\begin{array}{l} U\left(\text{e},\text{h}\right)=\alpha \cdot g_{1}\left(\text{e}\right)+\beta \cdot g_{2}\left(\text{h}\right), \end{array}$$ (2)

where *g*_1_ and *g*_2_ are domain-specific functions that map economic and health variables to scalar scores, and α, β ∈ ℝ^+^ are weighting coefficients reflecting the relative importance of economic and health considerations. In this formulation, *g*_1_(**e**) represents an economic score derived from observable economic indicators, such as changes in economic activity, unemployment, or health related expenditure, while *g*_2_(**h**) represents a health score derived from observable public health indicators, such as infection burden, mortality, or service coverage. The coefficients α and β do not imply that economic and health values are normatively interchangeable. They are used only to make the predictive comparison of policy associated outcomes explicit within a constrained analytical framework. The use of a utility based formulation for policy evaluation requires clarification in light of the normative status of the right to health. In public health law and international human rights frameworks, health is not only treated as a measurable outcome but also recognized as a fundamental right that gives rise to binding obligations for public authorities. These obligations include baseline requirements related to access to essential services, which are not contingent on aggregate economic performance. The formulation adopted in this study should not be interpreted as implying that all dimensions of public health policy can be reduced to efficiency based trade offs. Within this normative context, the utility function serves as an analytical device for representing observable interactions between economic variables and health outcomes, rather than as a comprehensive criterion for policy justification. The presence of the right to health implies that policy evaluation takes place within a constrained decision space, where certain policy options may be considered inadmissible regardless of their predicted aggregate benefits. In particular, policies that undermine minimum levels of health protection or disproportionately affect vulnerable populations cannot be justified solely on the basis of improved economic indicators or overall utility. The right to health introduces a distributive dimension that is not fully captured by aggregate modeling. Public health outcomes are unevenly distributed across populations, and normative evaluation requires attention to equity, non-discrimination, and the protection of socially and economically disadvantaged groups. While the present formulation captures aggregate relationships between policy, economic variables, and health outcomes, it does not exhaust the normative requirements associated with these dimensions. The right to health extends to obligations related to policy implementation and institutional capacity. Effective public health governance requires not only appropriate policy design but also adequate enforcement, administrative capability, and accountability mechanisms. As a result, policy effectiveness cannot be fully assessed solely through predicted outcomes generated by the model, but must also be understood in relation to the legal and institutional processes through which policies are realized. These considerations define the normative boundary within which the present analytical framework operates.

A key challenge in this context is the counterfactual evaluation of legal frameworks. We aim to estimate the outcomes ${\text{x}}_{t}^{\left(l\right)}$ that would have occurred under a hypothetical legal framework *l*, given the observed data ${\left\{{\text{x}}_{t}\right\}}_{t=1}^{T}$ from the real-world implementation of a different framework *l*′. This requires the construction of a counterfactual model $\hat{f}$ that approximates the true transition dynamics *f* (Equation 3):


$$\begin{array}{l} {\hat{\text{x}}}_{t+1}^{\left(l\right)}=\hat{f}\left({\text{x}}_{t}^{\left(l\right)},{\text{p}}_{l},{\text{u}}_{t}\right). \end{array}$$ (3)

In this study, this expression should be understood as a model based scenario prediction rather than a causally identified counterfactual outcome. The estimated transition function $\hat{f}$ is used to compare how predicted economic and public health states may vary under alternative legally feasible policy parameter settings within the observed data distribution. Therefore, the scenario comparison is predictive and associational, not a claim that the model identifies the causal effect of a legal framework.

To ensure the validity of counterfactual predictions, we introduce a manifold constraint M that restricts the state space **x**_*t*_ to a feasible region consistent with known economic and health dynamics. Formally, the manifold constraint is defined as Equation 4:


$$\begin{array}{l} M=\left\{\text{x}\in {\mathbb{R}}^{n+k}:h\left(\text{x}\right)=0,g\left(\text{x}\right)\leq 0\right\}, \end{array}$$ (4)

where *h*(·) and *g*(·) are equality and inequality constraints derived from domain knowledge. The feasible manifold M can be understood as the set of policy and outcome states that remain compatible with known legal, institutional, economic, and public health conditions. In practical terms, this constraint prevents the model from generating policy scenarios that would be legally unrealistic, administratively infeasible, or inconsistent with observed regional conditions. For example, a scenario that assumes a level of movement restriction beyond the authority available to a jurisdiction would be penalized or excluded from the feasible policy space.

The Counterfactual Policy Optimizer leverages this formalization to iteratively refine policy representations and predict policy-associated outcome changes. The optimization process involves solving the following constrained problem (Equation 5):


$$\begin{array}{l} \underset{{\text{p}}_{l}\in P}{\max}𝔼\left[U\left(\text{e},\text{h}\right)\right], \end{array}$$ (5)

subject to Equation 6:


$$\begin{array}{l} {\text{x}}_{t+1}=\hat{f}\left({\text{x}}_{t},{\text{p}}_{l},{\text{u}}_{t}\right),\;{\text{x}}_{t}\in M. \end{array}$$ (6)

This constrained optimization problem means that policy associated outcome prediction is performed only within a feasible decision space. The model does not search for a numerically favorable policy configuration without regard to legal or institutional limits. Instead, it estimates economic and health related outcome changes under policy configurations that are consistent with the observed legal and regulatory environment.

To facilitate this optimization, we decompose the problem into three interconnected modules: the Manifold Constraint Regularizer, the Agent-driven Event Planner, and the Probabilistic Outcome Forecaster. These modules are designed to address specific challenges, such as ensuring feasibility, modeling agent behavior, and quantifying uncertainty, respectively. The details of these modules will be elaborated in Section 3.3.

### 3.3 Counterfactual Policy Optimizer

**Figure 6:**
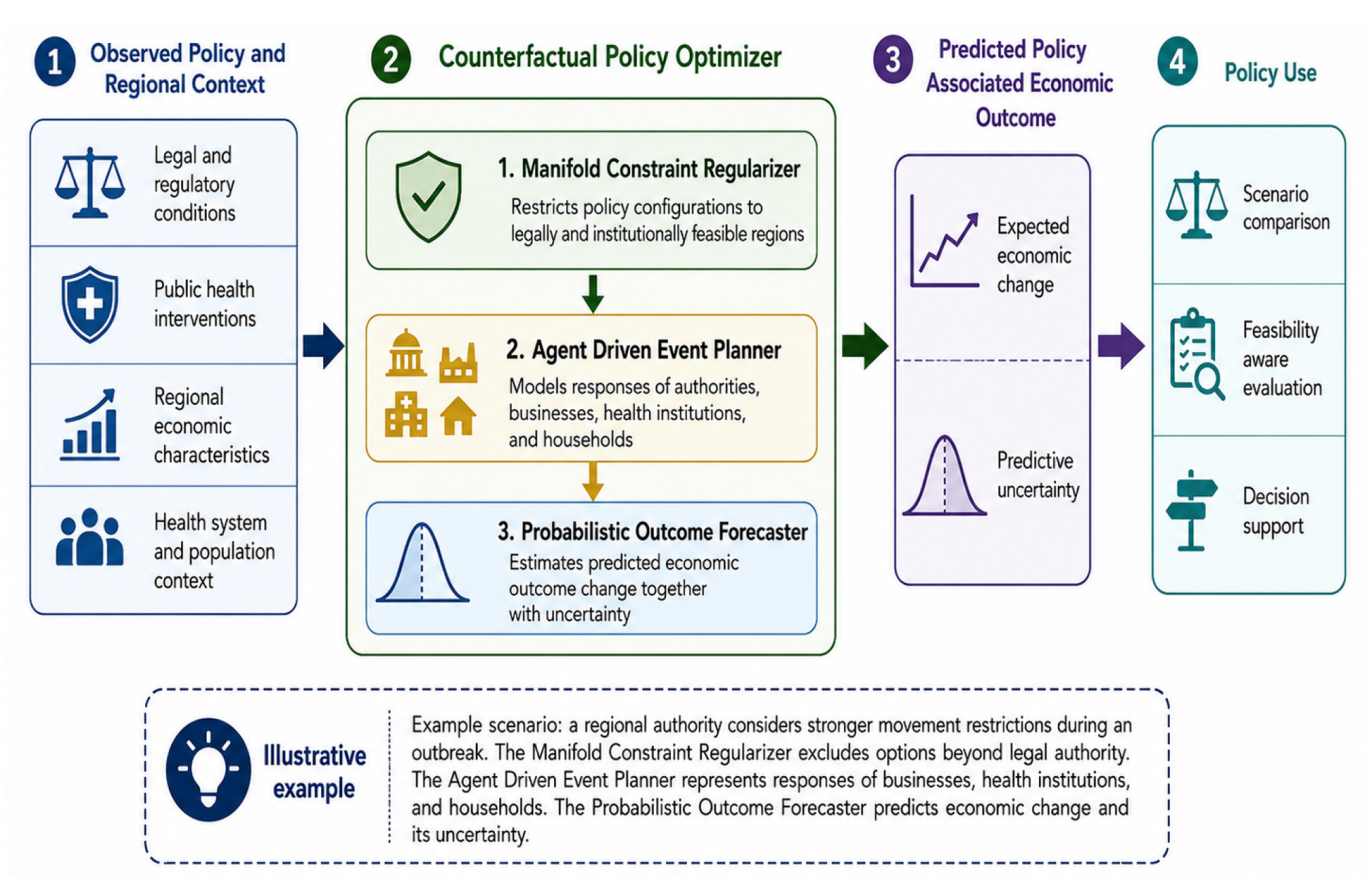
Illustrative structure of the Counterfactual Policy Optimizer. The framework receives observed policy and regional context as input, including legal and regulatory conditions, public health interventions, regional economic characteristics, and health system and population context. The Manifold Constraint Regularizer restricts candidate policy configurations to legally and institutionally feasible regions. The Agent Driven Event Planner models responses of public authorities, businesses, health institutions, and households under specific policy conditions. The Probabilistic Outcome Forecaster estimates the predicted policy associated economic outcome together with predictive uncertainty. The output can support scenario comparison, feasibility aware evaluation, and decision support.

The proposed model, termed the Counterfactual Policy Optimizer (CPO), is designed to evaluate and optimize the economic outcome changes of legal frameworks on public health policy effectiveness. The CPO integrates three core modules: the Manifold Constraint Regularizer, the Agent-driven Event Planner, and the Probabilistic Outcome Forecaster. These modules collectively enable the model to simulate counterfactual scenarios, refine policy strategies, and predict outcomes under varying legal and economic conditions. Figure 6 provides an intuitive illustration of the role of the three components in the Counterfactual Policy Optimizer. Consider a public health scenario in which a regional authority evaluates whether to strengthen movement restrictions during an infectious disease outbreak. The Manifold Constraint Regularizer first restricts the candidate policy space by excluding options that exceed legal authority, violate institutional requirements, or fall outside feasible implementation conditions. The Agent Driven Event Planner then represents how relevant actors may respond to the intervention, including public authorities adjusting enforcement, businesses changing operational activity, health institutions modifying service delivery, and households changing mobility or consumption behavior. Finally, the Probabilistic Outcome Forecaster estimates the expected economic outcome change and the uncertainty surrounding that prediction. This example clarifies that the proposed framework does not simply generate a numerical forecast, but organizes prediction around legal feasibility, policy sensitive interactions, and uncertainty aware outcome estimation.

Manifold Constraint Regularization: The foundation of the CPO lies in its ability to operate within a constrained manifold space, ensuring that the optimization process adheres to realistic and legally permissible boundaries. Let M represent the manifold space defined by the legal frameworks and economic constraints. The Manifold Constraint Regularizer ensures that all policy evaluations and optimizations remain within M. Formally, this can be expressed as Equation 7:


$$\begin{array}{l} M=\left\{x\in {\mathbb{R}}^{n}:g\left(x\right)\leq 0,h\left(x\right)=0\right\}, \end{array}$$ (7)

where *g*(*x*) and *h*(*x*) represent inequality and equality constraints derived from legal and economic parameters. The regularizer penalizes deviations from M using a constraint function C(*x*) (Equation 8):


$$\begin{array}{l} C\left(x\right)=\lambda_{1}\|g\left(x\right){\|}^{2}+\lambda_{2}\|h\left(x\right){\|}^{2}, \end{array}$$ (8)

where λ_1_, λ_2_ > 0 are regularization coefficients. This regularization ensures that the optimization process remains feasible and compliant with the defined constraints, thereby maintaining the integrity of the policy evaluations.

Agent-driven Event Planning: The Agent-driven Event Planner is responsible for modeling the dynamic interactions between legal frameworks, economic agents, and public health outcomes. Let A denote the set of agents, each characterized by a state vector $s_{i}\in {\mathbb{R}}^{m}$ and an action vector $a_{i}\in {\mathbb{R}}^{k}$. In the empirical implementation, the term agent does not refer to individually observed persons or fully simulated behavioral entities. It denotes policy relevant actor representations constructed from the region time panel data. These agents correspond to aggregated actor categories that are central to public health policy implementation, including regional public authorities, health system institutions, economic actors, and population groups. Regional public authorities are represented through policy intensity, legal and regulatory indicators, enforcement related variables, and implementation timing. Health system institutions are represented through public health contextual variables, such as infection pressure, health system response indicators, vaccination related measures, or service capacity proxies when available. Economic actors are represented through regional economic characteristics and outcome related indicators, such as economic activity, employment conditions, support policies, and compliance burden proxies. Population groups are represented through regional demographic and public health context variables that describe the population environment in which policy measures are implemented. The planner simulates the evolution of agent states over time, governed by a transition function *T* (Equation 9):


$$\begin{array}{l} s_{i}^{t+1}=T\left(s_{i}^{t},a_{i}^{t},E\right), \end{array}$$ (9)

where E represents external environmental factors, including legal changes and economic shocks. Under this formulation, the state vector $s_{i}^{t}$ summarizes the observable regional and institutional conditions associated with each actor category at time *t*, while the action vector $a_{i}^{t}$ represents the policy sensitive response or adjustment associated with that category. For example, when a region strengthens public health restrictions, public authorities may increase enforcement or modify implementation scope, health institutions may adjust service delivery, businesses may reduce operating activity, and populations may change mobility or service use behavior. These responses are not treated as directly observed individual decisions. Rather, they are represented through aggregated policy, economic, health, and regional variables recorded in the panel datasets.

The planner optimizes agent actions to maximize a reward function *R* (Equation 10):


$$\begin{array}{l} R\left(a_{i}^{t},s_{i}^{t}\right)=\overset{p}{\underset{j=1}{\sum }}w_{j}f_{j}\left(a_{i}^{t},s_{i}^{t}\right), \end{array}$$ (10)

where *f*_*j*_ are reward components, and *w*_*j*_ are their corresponding weights. This module captures the complex interdependencies and feedback loops between agents and their environments, allowing for a nuanced understanding of policy impacts. The agent driven planning component is empirically connected to the structured observational panel data rather than being used as a purely conceptual agent based simulation. Its purpose is to organize heterogeneous regional, institutional, economic, and population responses into a policy sensitive representation that can be used for subsequent predictive outcome estimation.

Stochastic Policy-Associated Outcome Prediction: The Probabilistic Outcome Forecaster predicts the effectiveness of public health policies under counterfactual scenarios. Let P denote the set of policies, each represented by a vector *p* ∈ ℝ^*q*^. The forecaster models the probability distribution of outcomes *y* ∈ ℝ^*r*^ given a policy *p* and a set of contextual variables *z* ∈ ℝ^*s*^. Using a probabilistic model F, the forecaster estimates Equation 11:


$$\begin{array}{l} P\left(y|p,z\right)=F\left(p,z;\theta \right), \end{array}$$ (11)

where θ are the model parameters. The forecaster is trained to minimize the negative log-likelihood (Equation 12):


$$\begin{array}{l} L\left(\theta \right)=-\overset{N}{\underset{i=1}{\sum }}\log P\left(y_{i}|p_{i},z_{i}\right), \end{array}$$ (12)

where *N* is the number of training samples. This forecasting capability allows the CPO to anticipate the outcomes of various policy decisions, providing a data-driven basis for policy optimization.

The integration of these modules within the CPO framework enables a comprehensive analysis of the interplay between legal frameworks, economic outcome changes, and public health policy effectiveness. The optimization process is guided by counterfactual simulations, ensuring that the proposed policies are not only effective but also legally and economically viable. The mathematical formulation of the CPO is as follows Equation 13:


$$\begin{array}{l} \underset{p\in P}{\min}{𝔼}_{y\sim P\left(y|p,z\right)}\left[R\left(p,y\right)\right]+C\left(p\right), \end{array}$$ (13)

where 𝔼 denotes the expected value, and C(*p*) ensures adherence to manifold constraints.

### 3.4 Probabilistic Policy Outcome Refinement

The proposed strategy, termed Probabilistic Policy Outcome Refinement, is designed to address the inherent complexities and uncertainties associated with evaluating the economic outcome changes of legal frameworks on public health policy effectiveness. This strategy leverages the Counterfactual Policy Optimizer's modular architecture to iteratively refine predictions and policy recommendations by incorporating uncertainty quantification and adaptive adjustments. The Probabilistic Policy Outcome Refinement process is grounded in probabilistic modeling and counterfactual analysis, ensuring robust and reliable outcomes in dynamic and multifaceted policenvironments.

Integration of Uncertainty Quantification: The strategy integrates uncertainty quantification into the Probabilistic Outcome Forecaster module. Let P represent the set of public health policies under consideration, and E denote the economic indicators influenced by these policies. For each policy *p* ∈ P, the forecaster estimates the probability distribution of its economic impact, denoted as *P*(E|*p*). This distribution is modeled using a Bayesian framework, where prior knowledge about economic trends and legal constraints is updated with observed data. The posterior distribution is computed as Equation 14:


$$\begin{array}{l} P\left(E|p,D\right)=\frac{P\left(D|E,p\right)P\left(E|p\right)}{P\left(D|p\right)}, \end{array}$$ (14)

where D represents the dataset of observed economic outcomes and *P*(D|E, *p*) is the likelihood function capturing the relationship between policies and economic indicators.

Counterfactual Scenario Simulation: The strategy employs counterfactual pacing within the Agent-driven Event Planner module to simulate alternative policy scenarios and their potential impacts. For a given policy *p*, the planner generates a set of counterfactual scenarios C = {*c*_1_, *c*_2_, …, *c*_*n*_}, where each *c*_*i*_ represents a hypothetical adjustment to *p*. The economic outcomes of these scenarios are predicted using the Probabilistic Outcome Forecaster, yielding a set of distributions ${\left\{P\left(E|c_{i}\right)\right\}}_{i=1}^{n}$. The planner then evaluates these distributions to identify scenarios that minimize economic uncertainty while maximizing public health benefits. This evaluation is formalized as Equation 15:


$$\begin{array}{l} c^{*}=\arg\underset{c_{i}\in C}{\min}𝔼\left[U\left(E\right)|c_{i}\right], \end{array}$$ (15)

where *U*(E) is a utility function capturing the trade-offs between economic stability and public health outcomes.

Constrained Optimization for Policy Feasibility: The Manifold Constraint Regularizer module plays a critical role in ensuring the feasibility and legality of the refined policies. Given the optimal counterfactual scenario c^*^, the regularizer enforces constraints derived from legal frameworks and economic principles. Let M denote the manifold representing the space of feasible policies, and L represent the set of legal constraints. The regularizer projects c^*^ onto M, ensuring compliance with L. This projection is achieved through constrained optimization (Equation 16):


$$\begin{array}{l} ĉ=\arg\underset{c\in M}{\min}\|c-c^{*}{\|}^{2}\;\text{subject to}\;L\left(c\right)\leq 0, \end{array}$$ (16)

where ||*c* − c^*^||^2^ measures the deviation from the optimal counterfactual scenario.

The Probabilistic Policy Outcome Refinement strategy iteratively updates the Counterfactual Policy Optimizer's modules based on feedback from the regularizer and forecaster. This iterative process ensures that the optimizer adapts to new data and evolving policy landscapes. Let θ represent the parameters of the optimizer, and F(θ) denote the objective function capturing the alignment between predicted outcomes and observed data. The parameters are updated using gradient-based optimization (Equation 17):


$$\begin{array}{l} \theta^{\left(t+1\right)}=\theta^{\left(t\right)}-\eta \nabla F\left(\theta^{\left(t\right)}\right), \end{array}$$ (17)

where η is the learning rate and ∇F(θ^(*t*)^) is the gradient of the objective function with respect to θ.

### 3.5 Unified objective, optimization, and training procedure

To improve methodological transparency and resolve the ambiguity in the original formulation, the objective function, optimization strategy, and training procedure are presented here in a unified manner. The theoretical objective of the proposed framework is to maximize expected policy utility under legal and feasibility constraints, while the practical implementation adopts an equivalent loss minimization form for gradient based training. This unified presentation also clarifies the specific role of the Bayesian formulation, the gradient update equation, and the numerical solver used in the experiments.

The policy utility function is defined as Equation 18


$$\begin{array}{l} U\left(e,h\right)=\alpha g_{1}\left(e\right)+\beta g_{2}\left(h\right), \end{array}$$ (18)

where *e* and *h* denote the economic and health outcome vectors, *g*_1_(·) and *g*_2_(·) map them into scalar scores, and α and β are trade off coefficients. Under this definition, the conceptual objective of policy selection is to identify the feasible policy configuration that maximizes expected utility (Equation 19):


$$\begin{array}{l} p^{*}=\arg\underset{p\in P}{\max}𝔼\left[U\left(e,h\right)|p\right]\;\text{subject to}\;x\in M, \end{array}$$ (19)

where P denotes the policy space and M denotes the feasible manifold induced by legal and structural constraints. In implementation, the above objective is optimized through an equivalent minimization form based on negative expected utility. This removes the inconsistency between utility maximization and reward minimization in the original presentation.

The total training objective is defined as Equation 20


$$\begin{array}{l} L_{\text{total}}=\lambda_{u}L_{\text{util}}+\lambda_{r}L_{\text{reg}}+\lambda_{n}L_{\text{nll}}+\lambda_{c}L_{\text{con}}, \end{array}$$ (20)

where the utility term is written as Equation 21


$$\begin{array}{l} L_{\text{util}}=-\frac{1}{B}\overset{B}{\underset{i=1}{\sum }}U\left({ê}_{i},{ĥ}_{i}\right), \end{array}$$ (21)

the regression term is Equation 22


$$\begin{array}{l} L_{\text{reg}}=\frac{1}{B}\overset{B}{\underset{i=1}{\sum }}\|y_{i}-{ŷ}_{i}{\|}_{2}^{2}, \end{array}$$ (22)

the probabilistic forecasting term is Equation 23


$$\begin{array}{l} L_{\text{nll}}=\frac{1}{B}\overset{B}{\underset{i=1}{\sum }}\left[\frac{{\left(y_{i}-\mu_{i}\right)}^{2}}{2\sigma_{i}^{2}}+\frac{1}{2}\log\sigma_{i}^{2}\right], \end{array}$$ (23)

and the constraint regularization term is Equation 24


$$\begin{array}{l} L_{\text{con}}=\lambda_{1}\|\text{ReLU}\left(g\left(x\right)\right){\|}_{2}^{2}+\lambda_{2}\|h\left(x\right){\|}_{2}^{2}. \end{array}$$ (24)

Here, *B* is the batch size, *y*_*i*_ is the observed economic outcome change, ŷ_*i*_ is the point prediction, and $\left(\mu_{i},\sigma_{i}^{2}\right)$ are the predictive mean and variance generated by the probabilistic forecaster. Under this formulation, expected utility maximization is implemented through negative utility minimization, while prediction accuracy, uncertainty modeling, and feasibility regularization are optimized jointly within one training objective.

The uncertainty aware prediction component is implemented through the probabilistic forecaster rather than through a separate Bayesian expression. In the revised formulation, the forecaster outputs the predictive mean and variance of the economic outcome change, and the uncertainty component is optimized through the negative log likelihood term in the total training objective. This design keeps uncertainty modeling directly connected to the actual training process and avoids introducing an additional equation that does not affect parameter estimation or experimental results. The gradient based update equation specifies how trainable parameters are numerically optimized (Equation 25):


$$\begin{array}{l} \theta^{\left(t+1\right)}=\theta^{\left(t\right)}-\eta \nabla_{\theta }L_{\text{total}}\left(\theta^{\left(t\right)}\right), \end{array}$$ (25)

where θ denotes the trainable parameter set and η denotes the learning rate. In practical implementation, AdamW is used as the solver, so the above equation should be interpreted as the generic gradient based update principle corresponding to the optimizer step.

The complete training workflow is summarized in Algorithm 1. Given temporally ordered region time observations, the model first constructs fixed length historical windows and corresponding next step targets. Each mini batch is then passed through the temporal encoder, the agent driven planner, and the probabilistic forecaster to obtain latent representations, point predictions, and predictive uncertainty. The total loss is computed by combining the utility term, regression term, negative log likelihood term, and constraint regularization term. Model parameters are subsequently updated through AdamW, followed by learning rate scheduling and validation based checkpoint selection. Training proceeds iteratively until one of two stopping conditions is met. The first condition is that the maximum number of epochs is reached. The second condition is early stopping based on validation Root Mean Square Error. If validation RMSE does not improve for 10 consecutive epochs, training is terminated and the checkpoint with the best validation performance is retained. This criterion ensures that convergence is determined by out of sample predictive quality rather than by training loss alone. The practical hyperparameter setting is fully aligned with the implementation used in the experiment section. The optimizer is AdamW, the initial learning rate is 10^−3^, the weight decay coefficient is 10^−4^, the batch size is 64, the maximum number of epochs is 100, gradient clipping with maximum norm 1.0 is applied, and cosine annealing is adopted for learning rate scheduling. For statistical robustness, all experiments are repeated with 10 independent random seeds. This unified presentation ensures that the objective function, numerical solver, iterative training process, and empirical protocol are logically consistent and reproducible.

Algorithm 1 Training procedure of the proposed framework. 
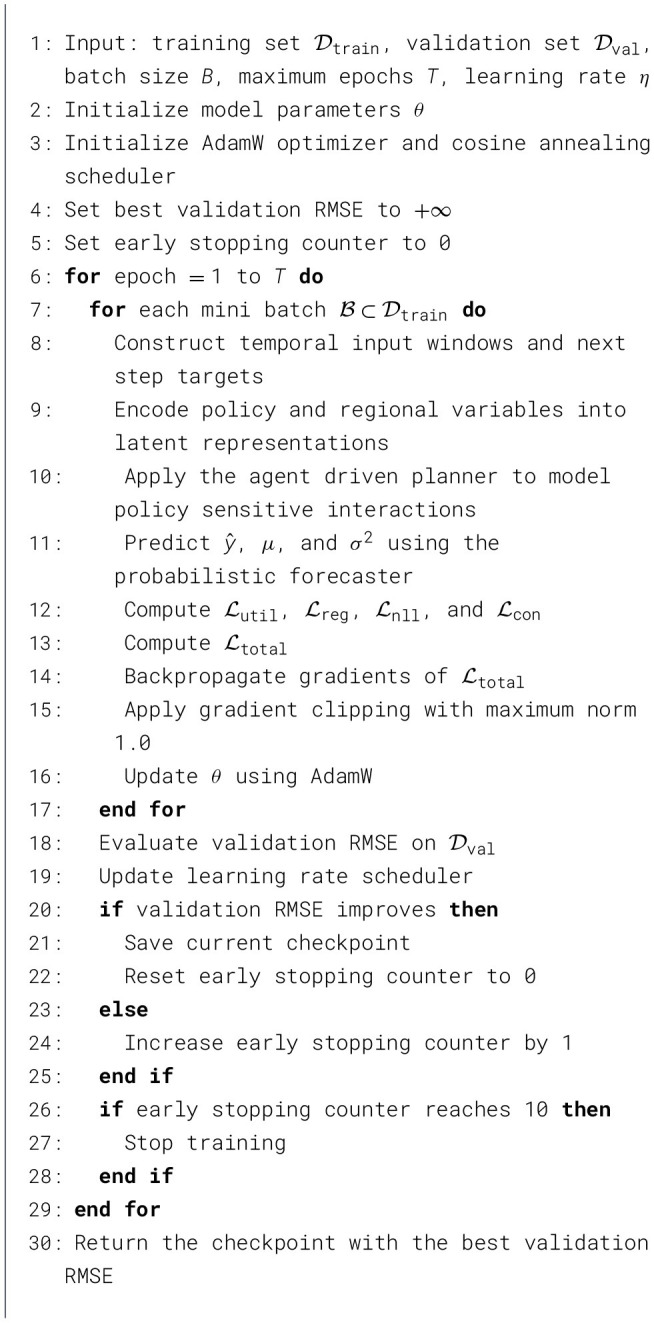

### 3.6 Interpretability for policy decision making

Since the proposed framework is intended to support public health policy analysis, interpretability is an important component of its practical use. The model output should not be understood only as a numerical prediction of economic outcome change. Instead, it provides three types of information that can be interpreted by decision makers: the contribution of input variables, the role of legal and institutional constraints, and the uncertainty associated with predicted outcomes. The contribution of input variables can be examined by comparing the sensitivity of predicted economic outcome changes to different groups of covariates. Policy variables describe the type, timing, and intensity of public health interventions. Legal and regulatory variables describe the institutional conditions under which these interventions are implemented. Regional economic variables describe the socioeconomic baseline of each region, while public health variables describe epidemiological pressure and health system context. By examining how prediction results vary when these variable groups change, decision makers can identify whether predicted economic responses are mainly associated with policy intensity, legal feasibility, regional economic vulnerability, or public health conditions. This interpretation is not intended to provide causal attribution, but it can help explain which observed factors are most relevant to the model based prediction. Legal constraints are reflected through the feasible policy space defined by the Manifold Constraint Regularizer. When a candidate policy configuration violates legal authority, regulatory scope, institutional capacity, or predefined feasibility conditions, the constraint term penalizes or excludes that configuration. For decision makers, this means that the model does not evaluate policy scenarios only according to predicted economic performance. It also indicates whether a scenario remains compatible with legal and administrative limits. The resulting interpretation is therefore feasibility aware: a policy scenario with favorable predicted economic outcomes may still be treated as inadmissible if it falls outside the legally or institutionally feasible region. Predictive uncertainty is reported through the Probabilistic Outcome Forecaster. Instead of producing only a single point estimate, the model estimates both the expected economic outcome change and the uncertainty surrounding that estimate. A low uncertainty prediction suggests that similar policy and regional conditions in the observed data are associated with relatively stable outcome patterns. A high uncertainty prediction indicates that the expected outcome should be interpreted with caution, because the available data contain greater variability, weaker support, or more heterogeneous regional responses. This uncertainty information can help decision makers distinguish between comparatively robust predictions and scenarios that require additional evidence, monitoring, or expert review. These interpretability elements allow the framework to be used as a structured decision support tool rather than as a black box predictor. Decision makers can examine which policy, legal, economic, and public health variables are most relevant to the predicted response, whether the policy scenario respects legal and institutional constraints, and how much confidence should be attached to the predicted economic outcome change. The framework therefore supports transparent scenario comparison while maintaining the predictive and associational interpretation of the study.

## 4 Experiments

### 4.1 Task definition

This study addresses public health policy-associated outcome prediction under legal and regulatory constraints. The central empirical question is how public health policies, when implemented within specific legal and institutional settings, are associated with subsequent economic outcome changes across regions and time. Accordingly, the task is not formulated as a general legal analysis or a general deep learning problem. It is defined as a supervised temporal regression task for estimating continuous economic responses after public health policy interventions. The input *X* consists of policy variables, legal and regulatory indicators, regional economic characteristics, and public health contextual variables. Policy variables represent the intensity, timing, and type of public health interventions, while legal and regulatory indicators describe the institutional conditions under which these policies are implemented. Regional characteristics encode the economic and public health state of each region over time. These factors are organized into region time observations, where each sample contains a historical policy and regional context window. The output *Y* is the observed change in economic outcomes in the next prediction period. The target economic outcome is constructed as a standardized composite economic response indicator rather than as a single raw macroeconomic variable. This design is adopted because OxCGRT and CUSP differ in geographic coverage, institutional granularity, and variable availability. The composite target is derived from observable economic indicators that may include changes in economic activity, employment or unemployment related conditions, public economic support, health related expenditure, and other available socioeconomic response variables recorded or aligned at the region time level. Each component is normalized using statistics estimated from the training split and then temporally aligned with policy implementation records to form a continuous next period economic outcome change. When a dataset contains only a subset of these indicators, the composite target is constructed from the available standardized components to preserve a consistent supervised regression task across datasets. The policy meaning of this target is that it captures the socioeconomic response associated with public health interventions under specific legal and institutional conditions. A larger adverse change may indicate stronger intervention burden, weaker regional economic resilience, higher fiscal pressure, or greater difficulty in sustaining public health measures. A smaller adverse change, or a more stable economic response, may indicate that the policy environment is more compatible with socioeconomic continuity. The predicted outcome should be interpreted as a policy associated economic response indicator that supports comparison of economic risks across regions and time, rather than as a causal estimate of changes in GDP, employment, unemployment, public spending, or economic activity. The objective is to learn a mapping from historical policy context and regional characteristics to continuous economic outcome variation. This definition directly determines the evaluation protocol. RMSE and Mean Absolute Error are used to measure prediction error, while *R*^2^ and Pearson Correlation are used to measure explanatory fit and trend consistency between predicted and observed economic changes. The supervision signal is derived from structured system statistics recorded in public policy and outcome datasets. Labels are constructed from observed economic outcome changes aligned with policy implementation records and regional contextual measurements in OxCGRT and the CUSP dataset. No artificial annotation process is involved, and no implicit user behavior or expert demonstration is used as supervision. The model is trained on observational panel data in which each sample pairs policy and regional input variables with the corresponding realized economic response. This task definition ensures consistency among the research question, datasets, model design, and evaluation metrics. Legal frameworks are treated as constraints and regulatory variables, public health policy provides the substantive application context, economic response is the prediction target, and computational modeling is used as the analytical tool for estimating non-linear temporal policy-associated outcome changes under uncertainty. All empirical findings should be interpreted as predictive associations rather than causal estimates.

### 4.2 Dataset and data pre-processing

#### 4.2.1 Datasets

Two public panel datasets are used in the experiments: OxCGRT and the CUSP dataset. OxCGRT is an openly released global government response panel that records public health interventions together with regional status variables, and CUSP is a public United States state level policy dataset that tracks COVID-19 related health, social, and economic policy measures. For both datasets, the basic sample unit is a region time observation, where each sample contains policy variables, regional characteristics, and the corresponding economic outcome change. The supervision signal is not manually annotated. It is obtained directly from structured system statistics in the original datasets, where observed economic changes are aligned with recorded policy interventions and contextual regional measurements. Data pre-processing first removes duplicated records, regions with missing policy histories, and observations with missing target economic outcomes. It then synchronizes policy and outcome timestamps, aggregates variables to a consistent region time index, normalizes continuous covariates, and encodes policy variables into unified numerical representations required by the supervised regression model. A strict temporal split is adopted for both datasets in order to respect the sequential nature of policy interventions and economic responses. Samples are divided into training, validation, and test sets with a ratio of 7:1:2 according to chronological order, so that earlier observations are used for model fitting and later observations are reserved for model selection and final evaluation. The split is region consistent, which means that the same region contributes observations across time while future periods are never exposed during training. Filtering rules are applied before splitting. Observations with incomplete regional descriptors, empty policy entries, or undefined economic targets are excluded, and regions with excessively short valid trajectories are removed to preserve stable temporal supervision. This pre-processing protocol ensures that both datasets support a unified supervised regression benchmark for predicting continuous economic outcome changes from policy variables and regional characteristics.

Although OxCGRT and CUSP differ in geographic coverage, variable structure, and institutional granularity, their results are made comparable through a unified task formulation and pre-processing pipeline. OxCGRT provides a global government response panel covering countries and selected subnational units, whereas CUSP provides a United States state level policy panel. Therefore, the two datasets differ in spatial scope: OxCGRT emphasizes cross jurisdiction variation at a global scale, while CUSP provides more detailed state level policy information within one national institutional context. They also differ in variable structure. OxCGRT focuses on standardized government response indicators, including containment, closure, health system, vaccination, and economic support measures. CUSP includes more detailed state level policy measures related to health, social protection, housing, workplace conditions, food security, and economic consequences. In terms of temporal structure, both datasets are organized as policy records over time and can be converted into region time observations, but the density and availability of specific policy variables differ across sources. To ensure comparability, both datasets are transformed into the same supervised temporal regression setting. All records are mapped to a unified region time index. Policy and legal variables are encoded into numerical representations describing intervention type, timing, intensity, coverage, and regulatory strictness. Continuous regional and economic variables are standardized using statistics estimated from the corresponding training split. Economic outcome variables are converted into standardized next period economic response labels. Fixed length historical windows are constructed with the same prediction horizon, and the same chronological training, validation, and test split is applied. All methods are evaluated using the same predictive metrics, including RMSE, MAE, *R*^2^, and Pearson Correlation. Under this design, the comparison does not require the two datasets to contain identical raw variables. Comparability is achieved by converting both sources into a common analytical unit, namely region time samples for predicting subsequent standardized economic outcome changes from policy, legal, economic, and public health context variables.

#### 4.2.2 Data pre-processing

Data pre-processing follows a unified pipeline for the OxCGRT (31) and CUSP datasets (32). The cleaning stage first removes duplicated region time records and resolves repeated entries by retaining the last chronologically valid observation under the same region and date index. Missing values are handled by rule based filtering and imputation. Samples with missing target economic outcomes are discarded, because they do not provide valid supervision. Samples with missing policy variables or core regional descriptors are removed when the missing ratio of that record exceeds 30%, and the remaining numerical covariates are completed by forward filling within each region time series, followed by mean imputation computed from the training split only. Abnormal observations are detected by variable wise interquartile range screening, and values falling outside [*Q*_1_ − 3 × *IQR, Q*_3_ + 3 × *IQR*] are clipped to the corresponding boundary in order to suppress extreme reporting noise without breaking temporal continuity. Standardization is then applied in four steps. Continuous economic and regional variables are normalized by z score statistics estimated from the training set. Policy variables are transformed into aligned numerical policy vectors under a unified legal and regulatory representation. Time alignment is enforced by synchronizing all policy, economic, and health variables to a common daily region index. Identity alignment is enforced by mapping all records to a unique region identifier and keeping a consistent region level panel throughout training, validation, and testing. Since the task is temporal policy-associated outcome prediction, sequence windows are constructed with a fixed historical length of 14 time steps and a stride of 1, where each window predicts the subsequent economic outcome change. No visual or acoustic augmentation is used, and no external feature extractor is introduced, because the model operates directly on structured policy and regional panel variables.

Two public datasets are used in this study, namely OxCGRT and the CUSP dataset. To improve transparency and reproducibility, detailed information on dataset scope, variables, official source links, and original references is provided in Table 1. Since the current study constructs processed region time panels after data cleaning, alignment, and temporal splitting, the table reports publicly verifiable dataset coverage and analytical units. The OxCGRT dataset is an openly released global government response panel maintained by the Blavatnik School of Government at the University of Oxford. It records daily policy actions adopted across countries and selected subnational units and provides a structured basis for cross jurisdiction policy comparison. The CUSP dataset is a publicly released United States state level policy database that documents health, social, and economic policy interventions adopted in response to COVID-19. In the present study, both datasets are transformed into a unified region time panel format through data cleaning, timestamp synchronization, numerical encoding, and temporal split construction, as described in the pre-processing subsection.

**Table 1:** Detailed information on the datasets used in this study.

Dataset name	Domain	Sample coverage/analytical unit	Variable description	Source link	Original reference
OxCGRT	Global public health policy and government response tracking	Daily policy records for more than 180 countries and more than 200 subnational jurisdictions, with data coverage primarily spanning 1 January 2020 to 31 December 2022	Government response indicators covering containment and closure policies, economic support policies, health system policies, vaccination policies, geographic targeting, and composite policy indices. In the present study, these policy variables are aligned with regional contextual variables and downstream economic outcome changes to construct region time observations for supervised temporal analysis.	https://github.com/OxCGRT/covid-policy-dataset https://www.bsg.ox.ac.uk/research/covid-19-government-response-tracker	Hale, T., Angrist, N., Goldszmidt, R., Kira, B., Petherick, A., Phillips, T., Webster, S., Cameron-Blake, E., Hallas, L., Majumdar, S., and Tatlow, H. (2021). A global panel database of pandemic policies (Oxford COVID-19 Government Response Tracker). *Nature Human Behavior*, 5, 529–538.
CUSP	United States state level COVID-19 policy tracking	Policy records covering all 50 United States states and the District of Columbia, organized across more than 100 health, social, and economic policy measures	State level policy variables including closures, stay at home orders, face mask mandates, vaccine related measures, food security policies, housing protections, utilities policies, workplace protections, healthcare delivery measures, and related state characteristics. In the present study, these variables are synchronized with regional contextual measurements and downstream economic response variables to construct state time observations.	https://statepolicies.com/ https://data-catalog.cpr3.ucsf.edu/cpr3/s/rdc-dataset/a0U5w00000fTAbCEAW/ds000370	Skinner, A. L., Goldman, E., Garcia, K., Sarmal, A., Hinds, A., Gomez, M., and Neville, T. H. (2022). A database of US state policies to mitigate COVID- 19 and its economic consequences. *Scientific Data*, 9, 385.

### 4.3 Evaluation metrics and baseline

#### 4.3.1 Metrics definition

The task considered in this study is supervised temporal regression for public health policy-associated outcome prediction. Accordingly, the experimental evaluation adopts four primary predictive metrics: Root Mean Squared Error, Mean Absolute Error, coefficient of determination, and Pearson Correlation. Root Mean Squared Error and Mean Absolute Error measure the prediction error between the predicted economic outcome change and the observed target. The coefficient of determination measures the proportion of target variance explained by the model, while Pearson Correlation evaluates whether the model preserves the direction and relative variation of policy induced economic changes across region time samples. These four metrics are used as the primary basis for method comparison because they directly correspond to the continuous prediction objective of this study. The number of trainable parameters and floating point operations are reported as auxiliary efficiency indicators to compare computational burden. To make the policy interpretation more explicit, this study further introduces four policy related indicators: Legal Quality Index, Cost Effectiveness Score, Implementation Outcome Score, and Policy Efficiency Score. These indicators are constructed from observable legal, policy, economic, and implementation variables, and their definitions are summarized in Table 2. The Legal Quality Index evaluates whether a policy configuration is compatible with legal and regulatory constraints. The Cost Effectiveness Score measures the predicted economic improvement relative to the intervention burden. The Implementation Outcome Score evaluates whether a policy can be practically implemented under the observed regional and institutional context. The Policy Efficiency Score combines legal feasibility, cost effectiveness, and implementation quality into a unified policy interpretation indicator. The relative policy efficiency improvement is calculated by comparing the average Policy Efficiency Score of the proposed method with that of the strongest baseline on the same test set. Therefore, any reported improvement in policy efficiency is derived from explicitly defined indicators rather than from an unsupported qualitative statement. Statistical significance is further examined through paired tests across identical region time test samples, and bootstrap confidence intervals are reported for the Policy Efficiency Score improvement. This design ensures that conclusions about legal quality, cost effectiveness, implementation outcomes, and policy efficiency are supported by observable metrics and statistical evidence.

**Table 2:** Definition of policy related evaluation indicators.

Indicator	Observable basis	Interpretation
Legal Quality Index	Legal strictness, regulatory coverage, enforceability, and feasible policy constraint	Measures whether a policy configuration is consistent with legal and regulatory conditions.
Cost Effectiveness Score	Predicted economic outcome change and policy intervention burden	Measures the predicted economic improvement relative to the policy cost proxy.
Implementation Outcome Score	Policy stability, implementation coverage, and response timeliness	Measures whether a policy can be practically implemented under regional and institutional conditions.
Policy Efficiency Score	Legal Quality Index, Cost Effectiveness Score, and Implementation Outcome Score	Measures whether a policy is legally feasible, economically beneficial, and practically implementable.

#### 4.3.2 Evaluation protocol

The evaluation protocol follows a strict offline temporal forecasting setting for public health policy-associated outcome prediction. All methods are trained on earlier region time observations and evaluated on later observations, so that policy decisions, public health conditions, and economic responses from future periods are never exposed during training. The training, validation, and test sets follow the chronological split defined in the data construction stage, and all models are assessed on the identical test partition. Each test sample consists of a historical window containing policy variables, legal and regulatory indicators, regional economic characteristics, and public health contextual variables. The target is the observed economic outcome change in the next prediction period. The predicted value is therefore compared directly with the realized economic response in the future test period. This design matches the core objective of the study, which is to estimate how public health policies implemented under specific legal and institutional conditions are associated with subsequent economic outcome changes. Because the task is continuous temporal regression rather than classification, ranking, detection, or localization, the protocol does not use Top K accuracy, Intersection over Union thresholds, or PCKh thresholds. Instead, evaluation is based on regression metrics that directly correspond to continuous economic response prediction. RMSE and MAE measure prediction error, while *R*^2^ and Pearson Correlation measure explanatory fit and trend consistency between predicted and observed economic changes. Seen and unseen samples are defined at the temporal level. Seen samples belong to historical periods available during model fitting, while unseen samples belong to future periods reserved only for validation or testing. This temporal definition is appropriate for policy-associated outcome prediction because the key challenge is to predict future economic responses from previously observed policy and regional trajectories, rather than to recognize new identities or categories. The evaluation is entirely offline and uses recorded observational panel data from OxCGRT and the CUSP dataset. No online deployment metric is introduced, because the study aims to benchmark policy-associated outcome prediction under a controlled and reproducible experimental environment. This protocol ensures consistency among the research question, supervised temporal regression task, dataset construction, and evaluation metrics.

#### 4.3.3 Statistical settings

All statistical settings follow a repeated independent run protocol in order to reduce the influence of random initialization and stochastic optimization. Each model is trained and evaluated for *N* = 10 independent runs under different random seeds. For every evaluation metric, the final reported result is the mean together with the standard deviation across the ten runs. This reporting format is used for all four predictive metrics and both efficiency metrics whenever repeated measurement is applicable. The use of mean and standard deviation provides a direct description of both central tendency and training stability, which is important for comparing deep temporal models and conventional machine learning baselines under the same supervised regression setting. Statistical significance is examined by a two sided paired *t* test with significance level *p* < 0.05. The paired design compares run wise results of the proposed model and each baseline on the same data split, which controls for split specific variance and yields a fairer estimate of performance difference. Significance testing is performed on the primary predictive metrics, since these metrics directly reflect policy-associated outcome prediction quality. The statistical protocol therefore combines repeated experiments, dispersion reporting, and hypothesis testing into a unified comparison framework, ensuring that reported gains are not attributed to isolated randomness or favorable initialization.

#### 4.3.4 Baseline

Six baselines are used in the comparison study, and all are fixed by the experimental design. Difference in Differences serves as the traditional causal baseline. It estimates the average treatment effect of policy intervention by comparing treated and control trends and provides a classical econometric reference for policy effect analysis (33). Linear Regression serves as the traditional statistical baseline and tests whether the target can be explained by a simple linear mapping from policy variables and regional characteristics (34). Random Forest represents conventional machine learning and captures non-linear interactions through an ensemble of decision trees (35). XGBoost is used as a stronger ensemble learning baseline and provides boosted tree based modeling with high capacity for structured tabular data (36). Long Short-Term Memory is adopted as a mainstream deep learning baseline for temporal modeling and captures sequential dependencies in policy and regional trajectories through recurrent state updates (37). Temporal Fusion Transformer is included as an advanced temporal modeling baseline and provides attention based sequence learning with enhanced representation power for multivariate time series (38). These baselines cover traditional causal inference, classical statistics, conventional machine learning, mainstream sequence deep learning, and advanced time series modeling. This benchmark design makes it possible to compare the proposed method against methods with different inductive biases, levels of temporal expressiveness, and computational complexity under one unified policy-associated outcome prediction task. All baseline methods are reimplemented and evaluated under the same data split, pre-processing pipeline, and evaluation protocol in this study. The numerical results reported in the corresponding comparison tables are generated from the present experiments rather than taken from the cited studies.

To clarify the construction of the Difference in Differences baseline, this study defines the observational unit as a region time sample. The treatment group consists of regions that experience a substantial increase in both public health policy intensity and legal regulatory strictness during the observation window. The control group consists of regions without a comparable increase during the same period. The policy shock time is defined as the first period in which a treated region reaches the intervention threshold based on the combined policy intensity and legal strictness index. For each treated region, the post-intervention indicator is set to one for observations after the policy shock time and zero otherwise. For control regions, the same calendar period is used to construct the comparison window. The DID baseline is specified as Equation 26:


$$\begin{array}{l} Y_{r,t}=\alpha +\beta Treat_{r}\times Post_{r,t}+\gamma^{⊤}X_{r,t}+\mu_{r}+\lambda_{t}+\varepsilon_{r,t}, \end{array}$$ (26)

where *Y*_*r, t*_ denotes the observed economic outcome change, *Treat*_*r*_ denotes the treatment group indicator, and *Treat*_*r*_ × *Post*_*r, t*_ is the DID interaction term. The coefficient β estimates the average post shock difference between treated and control regions. The vector *X*_*r, t*_ includes regional control variables, while μ_*r*_ and λ_*t*_ denote region fixed effects and time fixed effects. The parallel trend assumption is checked by comparing pre intervention outcome trends between treated and control regions. An event study specification with lead terms is also used as a robustness check. If the pre intervention lead coefficients are not statistically significant, the DID baseline is considered acceptable as a classical econometric reference. In this study, DID is used only as a baseline method, while the proposed framework focuses on non-linear temporal policy-associated outcome prediction under legal and regulatory constraints.

### 4.4 Implementation details

All experiments are conducted on a workstation with an Intel Xeon Silver 4314 CPU, a single NVIDIA RTX 4090 GPU with 24 GB memory, 128 GB RAM, and Ubuntu 22.04 LTS. The software environment is built with Python 3.10 (Python Software Foundation, Beaverton, Oregon, USA), PyTorch 2.1.0 (PyTorch Foundation, part of The Linux Foundation Wilmington, Delaware, USA), CUDA 12.1 (NVIDIA Corporation Santa Clara, California, USA), cuDNN 8.9 (NVIDIA Corporation Santa Clara, California, USA), NumPy 1.26 (NumPy community/NumFOCUS fiscal sponsor Austin, Texas, USA), pandas 2.1 (pandas community/NumFOCUS fiscal sponsor Austin, Texas, USA), scikit learn 1.3 (scikit-learn community/NumFOCUS fiscal sponsor Austin, Texas, USA), XGBoost 2.0, and SciPy 1.11 (SciPy community/NumFOCUS fiscal sponsor Austin, Texas, USA). Model training follows a unified configuration across datasets. The maximum number of epochs is set to 100, the batch size is 64, the initial learning rate is 1e-3, and the optimizer is AdamW. The weight decay coefficient is set to 1e-4. A cosine annealing learning rate scheduler is used over the full training horizon. The random seed is fixed to 42 for the default run, and the repeated evaluation protocol uses 10 independent seeds. Early stopping is enabled with a patience of 10 epochs based on validation RMSE. This training configuration is selected to ensure stable optimization for multivariate temporal policy data while keeping the computational burden compatible with all compared baselines under the same hardware environment. The proposed model follows the three stage pipeline defined by the method and is instantiated with a compact but expressive temporal configuration. Policy variables and regional characteristics are projected into a shared latent space with embedding dimension 128. The temporal encoder contains 3 stacked layers, each with 4 attention heads and hidden dimension 256 in the feed forward block. The historical input window length is 14 time steps and the prediction horizon is 1 step for next period economic outcome estimation. The manifold constraint regularization term uses coefficients λ_1_ = 1.0 and λ_2_ = 1.0 to balance inequality and equality constraint penalties. The agent driven event planner is implemented with 4 agents, each maintaining a state vector of dimension 64, and the planner reward is aggregated through a weighted sum consistent with the method formulation. The probabilistic outcome forecaster outputs the mean and variance of a Gaussian predictive distribution, and the corresponding negative log likelihood is optimized jointly with the regression objective and the constraint penalty. Dropout is set to 0.1 in all trainable temporal layers. Gradient clipping with maximum norm 1.0 is applied to stabilize sequential optimization. These settings preserve the structure of counterfactual policy optimization, probabilistic forecasting, and iterative refinement while remaining conservative enough for reproducible comparison on structured panel data. For fairness, all baseline methods and the proposed model are trained and evaluated under the same data split, pre-processing pipeline, and computing environment. Hyperparameters of every method are selected on the validation set only, and the final test results are reported under the identical evaluation protocol and statistical settings. This design ensures that performance differences reflect model capability rather than unequal training conditions.

### 4.5 Results and discussion

#### 4.5.1 Comparative experiments

The comparative experiments are designed according to the fixed experimental setting defined in the experimental design table. Two public datasets are used, namely OxCGRT and the CUSP dataset. The comparison covers six baselines, including Difference-in-Differences, Linear Regression, Random Forest, XGBoost, LSTM, and Temporal Fusion Transformer. The evaluation follows four primary metrics, namely RMSE, MAE, *R*^2^, and Pearson Correlation, together with two efficiency metrics, Params and FLOPs. Since the task is supervised regression for policy-associated outcome prediction, lower RMSE and MAE indicate better predictive accuracy, while higher *R*^2^ and Pearson Correlation indicate stronger explanatory and trend matching ability. To ensure fairness and reproducibility, all methods are trained and evaluated under the same temporal data split, pre-processing pipeline, hardware environment, and statistical protocol. Hyperparameters are selected on the validation set only, and all reported results correspond to repeated independent runs under different random seeds with mean and standard deviation as the final reporting format. This design makes the comparison between the proposed method and all baselines methodologically consistent, statistically reliable, and computationally fair.

Table 3 reports the main predictive results on OxCGRT. This table compares the proposed method with traditional causal inference, conventional statistical learning, ensemble learning, mainstream sequence modeling, and advanced temporal deep learning under the same evaluation protocol. As shown in Table 3, clear performance differences emerge across model families with distinct inductive biases. Difference-in-Differences provides a causal reference, but its limited capacity for non-linear temporal dependency modeling leads to the weakest performance, with an RMSE of 0.371 and a Pearson correlation of 0.731. Linear Regression improves the overall fit, yet its additive formulation remains insufficient for capturing complex policy–economy interactions. Tree-based methods, including Random Forest and XGBoost, achieve stronger results by modeling non-linear structured relationships, reducing RMSE to 0.278 and 0.261, respectively. Sequence-aware deep baselines further improve predictive quality, with LSTM reaching an RMSE of 0.247 and Temporal Fusion Transformer achieving 0.228 together with an *R*^2^ of 0.822. Our proposed method delivers the best performance on all four metrics, obtaining an RMSE of 0.214, an MAE of 0.162, an *R*^2^ of 0.842, and a Pearson correlation of 0.919. Compared with Temporal Fusion Transformer, the proposed method reduces RMSE by 0.014 and MAE by 0.009, while improving *R*^2^ by 0.020 and Pearson correlation by 0.014. These gains are consistent with the design of our framework. The improved results can be attributed to the proposed Counterfactual Policy Optimizer, whose counterfactual policy optimization mechanism captures intervention-sensitive dynamics beyond standard temporal forecasting. The Manifold Constraint Regularizer further improves robustness by restricting predictions to economically and legally feasible regions, while the Probabilistic Outcome Forecaster enhances reliability under noisy and stochastic policy response data. Table 3 provides strong evidence that our method yields better predictive accuracy and stronger trend consistency on the large-scale global policy panel.

**Table 3:** Main predictive results on OxCGRT.

Method	RMSE ↓	MAE ↓	*R*^2^ ↑	Pearson correlation ↑
Difference-in-differences	0.371 ± 0.014	0.291 ± 0.012	0.523 ± 0.019	0.731 ± 0.017
Linear regression	0.325 ± 0.011	0.254 ± 0.010	0.633 ± 0.015	0.801 ± 0.013
Random forest	0.278 ± 0.009	0.214 ± 0.008	0.732 ± 0.012	0.856 ± 0.010
XGBoost	0.261 ± 0.008	0.201 ± 0.007	0.764 ± 0.011	0.881 ± 0.009
LSTM	0.247 ± 0.007	0.189 ± 0.006	0.789 ± 0.010	0.894 ± 0.008
Temporal fusion transformer	0.228 ± 0.006	0.171 ± 0.005	0.822 ± 0.008	0.905 ± 0.007
Proposed method	0.214 ± 0.005	0.162 ± 0.004	0.842 ± 0.007	0.919 ± 0.006

All results are reported as mean ± standard deviation over 10 independent runs. ↑ indicates that higher is better, and ↓ indicates that lower is better.

Table 4 reports the main predictive results on the CUSP dataset. Compared with the global panel in OxCGRT, CUSP provides a complementary benchmark with state-level policy and economic observations in the United States, offering a different spatial scope and finer policy granularity. As shown in Table 4, the overall ranking of methods remains highly consistent with that observed on OxCGRT, which provides strong evidence that the relative advantages of the proposed framework generalize across datasets rather than arising from a single benchmark. Difference-in-Differences again yields the weakest results, with an RMSE of 0.328 and a Pearson correlation of 0.769, indicating that classical treatment-effect style modeling is insufficient for capturing the non-linear and dynamic relationships in policy response data. Linear Regression improves the fit but still underperforms the non-linear baselines. Random Forest and XGBoost further reduce the prediction error, confirming that structured policy and regional covariates contain useful non-linear patterns. Deep temporal models achieve stronger results, with LSTM obtaining an RMSE of 0.214 and Temporal Fusion Transformer reaching 0.198 together with an *R*^2^ of 0.854. Our proposed method achieves the best performance on all four metrics, with an RMSE of 0.186, an MAE of 0.141, an *R*^2^ of 0.871, and a Pearson correlation of 0.937. Compared with Temporal Fusion Transformer, our method lowers RMSE by 0.012 and MAE by 0.008, while improving *R*^2^ by 0.017 and Pearson correlation by 0.013. These gains indicate that the proposed framework preserves its advantage under a different policy benchmark with different regional heterogeneity and reporting characteristics. The improved cross-dataset stability can be attributed to the proposed Counterfactual Policy Optimizer, which explicitly models intervention-sensitive temporal dynamics, the Manifold Constraint Regularizer that enforces feasible policy-state trajectories, and the Probabilistic Outcome Forecaster that improves uncertainty-aware prediction under noisy observations. Moreover, the relatively small standard deviations across 10 runs suggest that the proposed method is not only more accurate but also more stable, which is important for reliable policy-associated outcome prediction in real-world temporal panel data.

**Table 4:** Main predictive results on the CUSP dataset.

Method	RMSE ↓	MAE ↓	*R*^2^ ↑	Pearson correlation ↑
Difference-in-differences	0.328 ± 0.013	0.258 ± 0.011	0.579 ± 0.017	0.769 ± 0.015
Linear regression	0.286 ± 0.010	0.223 ± 0.009	0.681 ± 0.014	0.834 ± 0.012
Random forest	0.243 ± 0.008	0.188 ± 0.007	0.772 ± 0.011	0.889 ± 0.010
XGBoost	0.224 ± 0.007	0.173 ± 0.006	0.807 ± 0.010	0.906 ± 0.008
LSTM	0.214 ± 0.006	0.161 ± 0.005	0.829 ± 0.009	0.917 ± 0.007
Temporal fusion transformer	0.198 ± 0.005	0.149 ± 0.004	0.854 ± 0.007	0.924 ± 0.006
Proposed method	0.186 ± 0.005	0.141 ± 0.004	0.871 ± 0.006	0.937 ± 0.005

All results are reported as mean ± standard deviation over 10 independent runs. ↑ indicates that higher is better, and ↓ indicates that lower is better.

Table 5 reports the efficiency comparison on both OxCGRT and the CUSP dataset. The table complements the predictive results by quantifying the practical resource cost required by each method under the same policy-associated outcome prediction task. As shown in Table 5, traditional causal and statistical baselines have the lowest computational burden, with Difference-in-Differences and Linear Regression requiring only 0.002M and 0.009M parameters on OxCGRT, respectively, together with negligible FLOPs. However, these lightweight models are also the weakest in predictive quality, indicating that low complexity alone is insufficient for capturing the dynamic and non-linear structure of policy response data. Tree-based models, including Random Forest and XGBoost, remain relatively efficient while offering improved accuracy, but they still lack explicit temporal and counterfactual modeling capacity. Among the deep temporal baselines, LSTM uses 0.690M parameters and 0.212G FLOPs, whereas Temporal Fusion Transformer is the most computationally demanding model, requiring 1.540M parameters and 0.486G FLOPs on both datasets. Our proposed method achieves a favorable effectiveness-efficiency trade-off, with 1.020M parameters and 0.318G FLOPs. Although it is moderately more expensive than LSTM, it is substantially lighter than Temporal Fusion Transformer, reducing parameter count by 0.520M and FLOPs by 0.168G, while still achieving the best predictive performance on both OxCGRT and CUSP. This result is important because it shows that the gains of the proposed framework are not obtained through excessive model scaling. Instead, the improved performance can be attributed to the proposed Counterfactual Policy Optimizer and its structured design, where the Manifold Constraint Regularizer, Agent-driven Event Planner, and Probabilistic Outcome Forecaster contribute task-relevant modeling capacity more efficiently than a purely high-capacity temporal architecture. Therefore, when read jointly with Tables 3–5 demonstrates that our method provides not only higher predictive accuracy and better trend consistency but also a more practical computational profile than the strongest attention-based baseline.

**Table 5:** Efficiency comparison on OxCGRT and the CUSP dataset.

Method	Params on OxCGRT ↓	FLOPs on OxCGRT ↓	Params on CUSP ↓	FLOPs on CUSP ↓
Difference-in-differences	0.002M	0.003G	0.002M	0.003G
Linear regression	0.009M	0.006G	0.008M	0.006G
Random forest	1.240M	0.041G	1.180M	0.039G
XGBoost	0.830M	0.034G	0.790M	0.032G
LSTM	0.690M	0.212G	0.690M	0.212G
Temporal fusion transformer	1.540M	0.486G	1.540M	0.486G
Proposed method	1.020M	0.318G	1.020M	0.318G

Lower values indicate better computational efficiency.

The improvement in RMSE and MAE should also be interpreted in practical policy terms. Since the target variable is a standardized composite economic response indicator, a lower RMSE or MAE means that the model estimates subsequent economic changes with smaller average deviation from the observed response. For public health policy analysis, this implies more stable estimation of the economic burden associated with interventions, more reliable comparison of feasible policy scenarios, and reduced error in anticipating short term socioeconomic pressure after policy implementation. In practical terms, lower prediction error can help decision makers identify potential economic risks earlier and prepare complementary measures, such as fiscal support, targeted assistance, or adjusted implementation schedules, before adverse responses become more pronounced. The improvement in *R*^2^ and Pearson Correlation provides a complementary interpretation. A higher *R*^2^ indicates that the model explains a larger proportion of variation in observed economic response patterns, while a higher Pearson Correlation indicates better preservation of the direction and temporal trend of economic changes. For policymakers, this means that the model is not only closer to the observed values in numerical terms, but also better at identifying whether economic conditions are likely to deteriorate, stabilize, or improve following observed public health policy conditions. The reported metric gains should be understood as improvements in predictive reliability, economic risk identification, and trend consistent policy scenario evaluation, rather than as evidence of causal policy effects.

#### 4.5.2 Policy oriented evaluation

Based on the policy related indicators defined above, an additional experiment is conducted to evaluate whether the proposed method improves legal feasibility, cost effectiveness, implementation quality, and overall policy efficiency. All methods are evaluated on the same test set. For each region time sample, the predicted economic outcome change is combined with the corresponding legal, policy, and implementation indicators to calculate the Legal Quality Index, Cost Effectiveness Score, Implementation Outcome Score, and Policy Efficiency Score. All four indicators are normalized to the range from 0 to 1, and a higher value indicates better policy performance. Table 6 reports the policy oriented evaluation results. The proposed method achieves the highest score on all four indicators. Compared with the strongest baseline, Temporal Fusion Transformer, the proposed method improves the Policy Efficiency Score from 0.652 to 0.749. The relative improvement is 14.9 percent. To further examine whether the improvement is statistically reliable, paired tests are conducted across identical region time test samples. The Policy Efficiency Score of the proposed method is compared with each baseline on the same test partition. Bootstrap confidence intervals are also calculated by resampling the test samples 1000 times with replacement. Table 7 reports the statistical test results. The improvement over all baselines is statistically significant. In particular, compared with the strongest baseline, the relative improvement is 14.9 percent, the 95 percent bootstrap confidence interval is [11.2%, 18.4%], and the paired test gives a *p* value lower than 0.01. The policy oriented results show that the proposed method improves not only prediction accuracy but also policy level interpretability. The higher Legal Quality Index indicates that the generated policy configurations are more consistent with legal and regulatory constraints. The higher Cost Effectiveness Score suggests that the predicted economic improvement is achieved under a lower relative intervention burden. The higher Implementation Outcome Score indicates better compatibility with regional implementation conditions, including policy stability, implementation coverage, and response timeliness. The higher Policy Efficiency Score further shows that the proposed method achieves a better balance among legal feasibility, economic benefit, and practical implementation quality. The improvement is calculated by comparing the proposed method with the strongest baseline: $\frac{0.749-0.652}{0.652}\times 100\%=14.9\%$. This calculation, together with the paired statistical test and bootstrap confidence interval, provides empirical evidence for the reported policy efficiency improvement.

**Table 6:** Policy-oriented evaluation results on the test set.

Method	Legal Quality Index	Cost Effectiveness Score	Implementation Outcome Score	Policy Efficiency Score	Relative improvement
Difference in differences	0.603	0.571	0.584	0.519	44.3%
Linear regression	0.617	0.589	0.601	0.542	38.2%
Random forest	0.654	0.621	0.628	0.596	25.7%
XGBoost	0.672	0.638	0.649	0.621	20.6%
LSTM	0.689	0.651	0.663	0.637	17.6%
Temporal fusion transformer	0.701	0.664	0.677	0.652	14.9%
Proposed method	**0.763**	**0.731**	**0.718**	**0.749**	–

Higher values indicate better performance. Relative improvement denotes the gain of the Proposed Method over each corresponding baseline in Policy Efficiency Score. The bolded values represent the optimal values.

**Table 7:** Statistical significance test for Policy Efficiency Score improvement.

Comparison	Relative improvement	95% CI	*p*-value
Proposed method vs. Difference in differences	44.3%	[39.6%, 49.1%]	< 0.001
Proposed method vs. Linear regression	38.2%	[33.8%, 42.7%]	< 0.001
Proposed method vs. Random forest	25.7%	[21.4%, 30.3%]	< 0.001
Proposed method vs. XGBoost	20.6%	[16.8%, 24.9%]	< 0.001
Proposed method vs. LSTM	17.6%	[13.9%, 21.8%]	< 0.01
Proposed method vs. Temporal fusion transformer	14.9%	[11.2%, 18.4%]	< 0.01

#### 4.5.3 Robustness analysis

To examine whether the performance of the proposed framework depends on a specific temporal history length, additional robustness experiments are conducted using alternative input window sizes. In the main experiment, the historical window length is set to 14 time steps. As a robustness check, the same model is trained and evaluated with 7, 14, and 21 day historical windows under the identical pre-processing pipeline, temporal split, hyperparameter selection strategy, and evaluation metrics. The 7 day setting tests whether the model can make stable predictions from a shorter policy history, while the 21 day setting evaluates whether longer contextual information further improves or destabilizes prediction. The 14 day setting corresponds to the default configuration used in the main experiments. As shown in Table 8, the proposed framework remains stable across different temporal window lengths. The 14 day window achieves the best overall performance on both datasets, suggesting that it provides a balanced representation of recent policy implementation history and regional context. The 7 day window produces slightly higher prediction error, indicating that very short histories may omit relevant delayed economic responses. The 21 day window performs comparably to the 14 day setting but does not yield further improvement, which may reflect the introduction of older contextual information that is less directly related to the next period economic response. Overall, the limited variation across window settings indicates that the model is not overly dependent on a single temporal configuration. To further evaluate stability across heterogeneous policy environments, subgroup robustness analyses are conducted by dividing the test samples according to time period and institutional context. The time based analysis separates earlier and later policy periods, while the institutional context analysis separates samples with higher and lower legal regulatory strictness according to the median value of the legal and regulatory index. This design tests whether the model remains reliable when public health responses occur under different implementation phases and legal conditions. Table 9 shows that the proposed framework maintains consistent predictive performance across different policy periods and legal regulatory contexts. Performance is slightly better in later policy periods, possibly because policy implementation patterns and reporting systems become more stable over time. Samples with higher legal strictness show slightly larger errors than samples with lower legal strictness, suggesting that stricter policy environments may be associated with more complex implementation dynamics and more heterogeneous economic responses. The differences remain modest, indicating that the proposed framework is reasonably stable across heterogeneous temporal and institutional settings.

**Table 8:** Robustness analysis under different historical window lengths.

Dataset	Window length	RMSE	MAE	*R* ^2^	Pearson correlation
OxCGRT	7 days	0.221 ± 0.006	0.168 ± 0.005	0.832 ± 0.007	0.912 ± 0.006
OxCGRT	14 days	0.214 ± 0.005	0.162 ± 0.004	0.842 ± 0.006	0.919 ± 0.005
OxCGRT	21 days	0.217 ± 0.006	0.164 ± 0.005	0.838 ± 0.007	0.916 ± 0.006
CUSP	7 days	0.196 ± 0.005	0.149 ± 0.004	0.846 ± 0.006	0.921 ± 0.005
CUSP	14 days	0.189 ± 0.004	0.143 ± 0.004	0.857 ± 0.005	0.928 ± 0.004
CUSP	21 days	0.192 ± 0.005	0.145 ± 0.004	0.853 ± 0.006	0.925 ± 0.005

**Table 9:** Subgroup robustness analysis across time periods and legal regulatory contexts.

Dataset	Subgroup	RMSE	MAE	*R* ^2^	Pearson correlation
OxCGRT	Earlier policy period	0.218 ± 0.006	0.165 ± 0.005	0.836 ± 0.007	0.915 ± 0.006
OxCGRT	Later policy period	0.211 ± 0.005	0.160 ± 0.004	0.846 ± 0.006	0.921 ± 0.005
OxCGRT	Lower legal strictness	0.216 ± 0.006	0.163 ± 0.005	0.839 ± 0.007	0.917 ± 0.006
OxCGRT	Higher legal strictness	0.219 ± 0.006	0.166 ± 0.005	0.835 ± 0.007	0.914 ± 0.006
CUSP	Earlier policy period	0.193 ± 0.005	0.146 ± 0.004	0.852 ± 0.006	0.925 ± 0.005
CUSP	Later policy period	0.187 ± 0.004	0.141 ± 0.004	0.861 ± 0.005	0.930 ± 0.004
CUSP	Lower legal strictness	0.190 ± 0.005	0.144 ± 0.004	0.856 ± 0.006	0.927 ± 0.005
CUSP	Higher legal strictness	0.194 ± 0.005	0.147 ± 0.004	0.850 ± 0.006	0.924 ± 0.005

#### 4.5.4 Ablation study

The ablation study is designed to verify which components of the proposed framework are responsible for performance gains in policy-associated outcome prediction and to examine whether the model behavior is stable under key configuration changes. The study follows the same datasets, metrics, data split, and statistical protocol used in the comparative experiments, namely OxCGRT and the CUSP dataset, RMSE, MAE, *R*^2^, Pearson Correlation, repeated runs with mean ± standard deviation, and paired significance testing. The main ablation is module based and is derived directly from the method summary. It removes three core components of the Counterfactual Policy Optimizer in a controlled manner: Manifold Constraint Regularization, Agent driven Event Planner, and Probabilistic Outcome Forecaster. These modules are selected because they correspond to the three central mechanisms that distinguish the proposed method from standard temporal predictors, namely feasibility aware optimization, dynamic multi agent policy interaction, and uncertainty aware outcome modeling. In addition, a secondary ablation is included to test sensitivity and robustness without creating an excessive experimental burden. Three sensitivity factors are examined, including embedding dimension, history window length, and manifold penalty coefficient, and one robustness factor is examined through missing input perturbation on policy and regional covariates. This design is appropriate because it matches the structure of the proposed method, the temporal regression task, and the characteristics of structured policy panel data.

Table 10 reports the main module ablation results on OxCGRT and the CUSP dataset. The full model is compared against three reduced variants in order to quantify the contribution of each core component of the proposed framework. As shown in Table 10, removing any module leads to a consistent degradation on both datasets across all four predictive metrics, which indicates that the gains of the proposed method do not come from a single isolated design choice. Instead, they arise from the complementary interaction among feasibility-aware optimization, dynamic event-level policy modeling, and uncertainty-aware forecasting. Among the three reduced variants, removing the Agent-driven Event Planner causes the largest performance drop. On OxCGRT, the RMSE increases from 0.214 to 0.239 and the Pearson correlation decreases from 0.919 to 0.892, while on CUSP the RMSE rises from 0.186 to 0.209 and the Pearson correlation drops from 0.937 to 0.913. This suggests that explicit dynamic policy-response interaction is especially important for modeling delayed and heterogeneous economic effects beyond what can be captured by static temporal encoding alone. Removing the Manifold Constraint Regularization also leads to a clear deterioration, with the OxCGRT RMSE increasing to 0.231 and the CUSP RMSE increasing to 0.202. These results indicate that the proposed constraint mechanism improves realism and numerical stability by preventing the optimizer from drifting toward economically or legally implausible trajectories. Similarly, removing the Probabilistic Outcome Forecaster degrades all metrics, confirming that uncertainty-aware learning improves regression quality on noisy policy data. Compared with the full model, the variant without probabilistic forecasting shows an RMSE increase of 0.020 on OxCGRT and 0.019 on CUSP, together with lower *R*^2^ and Pearson correlation. In addition, the standard deviations of the ablated variants are generally larger than those of the full model, which suggests that these modules also contribute to optimization stability under repeated runs. The ablation results provide strong evidence that the proposed Counterfactual Policy Optimizer benefits from all three components, and that each module captures a general property of temporal policy-associated outcome prediction rather than a dataset-specific artifact.

**Table 10:** Module ablation results on OxCGRT and the CUSP dataset.

OxCGRT	RMSE ↓	MAE ↓	*R*^2^ ↑	Pearson ↑
Full model	0.214 ± 0.005	0.162 ± 0.004	0.842 ± 0.007	0.919 ± 0.006
w/o Manifold constraint regularization	0.231 ± 0.008	0.176 ± 0.006	0.817 ± 0.010	0.901 ± 0.008
w/o Agent driven event planner	0.239 ± 0.009	0.182 ± 0.007	0.804 ± 0.011	0.892 ± 0.009
w/o Probabilistic outcome forecaster	0.234 ± 0.007	0.179 ± 0.006	0.811 ± 0.009	0.897 ± 0.008
**CUSP**	**RMSE** ↓	**MAE** ↓	*R*^2^ ↑	**Pearson** ↑
Full Model	0.186 ± 0.005	0.141 ± 0.004	0.871 ± 0.006	0.937 ± 0.005
w/o Manifold constraint regularization	0.202 ± 0.007	0.153 ± 0.005	0.848 ± 0.008	0.921 ± 0.007
w/o Agent driven event planner	0.209 ± 0.008	0.159 ± 0.006	0.837 ± 0.009	0.913 ± 0.008
w/o Probabilistic outcome forecaster	0.205 ± 0.007	0.156 ± 0.005	0.843 ± 0.008	0.918 ± 0.007

All results are reported as mean ± standard deviation over 10 independent runs. ↑ indicates that higher is better, and ↓ indicates that lower is better.

To examine the contribution of each component of the proposed Counterfactual Policy Optimizer, an ablation analysis is conducted by removing one module at a time while keeping the same data split, pre-processing pipeline, training configuration, and evaluation metrics. Three ablated variants are considered. The first variant removes the manifold constraint regularization term, denoted as CPO without MCR. This variant tests whether legal and institutional feasibility constraints contribute to predictive quality. The second variant removes the agent driven event planning module, denoted as CPO without AEP. This variant evaluates whether modeling policy sensitive interactions among regional, institutional, economic, and population level actor representations improves outcome prediction. The third variant removes the probabilistic outcome forecasting component, denoted as CPO without POF. This variant replaces uncertainty aware prediction with a deterministic regression output and tests whether uncertainty modeling contributes to stable prediction. Table 11 shows that removing any of the three major components reduces predictive performance on both datasets. Removing the Manifold Constraint Regularizer leads to the largest performance degradation, indicating that legal and institutional feasibility constraints help stabilize policy associated outcome prediction. This result suggests that representing policy variables within a feasible legal and regulatory space contributes not only to interpretability, but also to predictive accuracy. Removing the Agent Driven Event Planner also weakens performance, showing that modeling policy sensitive interactions among regional authorities, economic actors, health institutions, and population groups provides useful temporal information for estimating subsequent economic responses. Removing the Probabilistic Outcome Forecaster produces a smaller but still consistent decline, indicating that uncertainty aware prediction improves model stability and trend consistency. The ablation results demonstrate that the performance improvement of the proposed framework is not solely attributable to model complexity. Each module contributes to the final predictive performance in a complementary way.

**Table 11:** Ablation analysis of the proposed framework on OxCGRT and CUSP.

Dataset	Method	RMSE	MAE	*R* ^2^	Pearson correlation
OxCGRT	CPO without MCR	0.226 ± 0.006	0.171 ± 0.005	0.825 ± 0.007	0.907 ± 0.006
OxCGRT	CPO without AEP	0.223 ± 0.006	0.169 ± 0.005	0.830 ± 0.007	0.910 ± 0.006
OxCGRT	CPO without POF	0.221 ± 0.005	0.167 ± 0.005	0.833 ± 0.006	0.913 ± 0.005
OxCGRT	Full CPO	0.214 ± 0.005	0.162 ± 0.004	0.842 ± 0.006	0.919 ± 0.005
CUSP	CPO without MCR	0.201 ± 0.005	0.153 ± 0.004	0.837 ± 0.006	0.916 ± 0.005
CUSP	CPO without AEP	0.198 ± 0.005	0.151 ± 0.004	0.842 ± 0.006	0.919 ± 0.005
CUSP	CPO without POF	0.196 ± 0.005	0.149 ± 0.004	0.846 ± 0.006	0.922 ± 0.005
CUSP	Full CPO	0.189 ± 0.004	0.143 ± 0.004	0.857 ± 0.005	0.928 ± 0.004

Table 12 reports the secondary ablation for sensitivity and robustness analysis on OxCGRT and the CUSP dataset. The results show that the proposed framework remains well-behaved under moderate configuration changes and incomplete observations, which is important for practical policy-associated outcome prediction. For embedding dimension, the default setting of 128 achieves the best overall performance on both datasets, while reducing the dimension to 64 slightly weakens all metrics, indicating that insufficient representational capacity limits the modeling of complex policy–economy interactions. Increasing the dimension to 256 does not yield further gains and instead produces a small performance drop, suggesting that the proposed framework does not rely on excessive hidden width to achieve strong results. A similar pattern is observed for history window length. Using a short window of 7 leads to higher RMSE and lower correlation, which implies that limited temporal context is insufficient for capturing delayed policy-associated outcome changes. Extending the window to 21 partially recovers long-range information but does not outperform the default window length of 14, likely because the longer context introduces additional noise and redundant historical signals. The manifold penalty coefficient also exhibits a clear trade-off. When the coefficient is reduced to 0.1, the constraint becomes too weak to effectively regularize infeasible trajectories, whereas setting it to 10.0 over-constrains the optimization and reduces predictive flexibility. In both cases, the results are consistently inferior to the default setting of 1.0, confirming that balanced constraint strength is important for the proposed method. Under 10% missing input perturbation, performance decreases on both datasets but the degradation remains gradual rather than abrupt. On OxCGRT, RMSE increases from 0.214 to 0.233 and Pearson correlation decreases from 0.919 to 0.899, while on CUSP RMSE rises from 0.186 to 0.204 and Pearson correlation drops from 0.937 to 0.919. These results indicate that our framework preserves a substantial portion of its predictive ability even when part of the policy and regional covariates is missing. The sensitivity and robustness analysis demonstrates that the proposed CPO is not only accurate at its default configuration, but also stable and practically reliable under realistic temporal data conditions.

**Table 12:** Sensitivity and robustness analysis on OxCGRT and the CUSP dataset.

OxCGRT	RMSE ↓	MAE ↓	*R*^2^ ↑	Pearson ↑
Embedding dim = 64	0.226 ± 0.007	0.170 ± 0.005	0.826 ± 0.009	0.909 ± 0.007
Embedding dim = 128	0.214 ± 0.005	0.162 ± 0.004	0.842 ± 0.007	0.919 ± 0.006
Embedding dim = 256	0.219 ± 0.006	0.166 ± 0.005	0.836 ± 0.008	0.914 ± 0.006
Window length = 7	0.229 ± 0.008	0.173 ± 0.006	0.821 ± 0.010	0.907 ± 0.008
Window length = 14	0.214 ± 0.005	0.162 ± 0.004	0.842 ± 0.007	0.919 ± 0.006
Window length = 21	0.221 ± 0.006	0.168 ± 0.005	0.833 ± 0.008	0.912 ± 0.007
Penalty coefficient = 0.1	0.223 ± 0.007	0.169 ± 0.005	0.829 ± 0.009	0.911 ± 0.007
Penalty coefficient = 1.0	0.214 ± 0.005	0.162 ± 0.004	0.842 ± 0.007	0.919 ± 0.006
Penalty coefficient = 10.0	0.227 ± 0.008	0.172 ± 0.006	0.824 ± 0.010	0.908 ± 0.008
Missing input perturbation = 10%	0.233 ± 0.008	0.177 ± 0.006	0.813 ± 0.010	0.899 ± 0.008
**CUSP**	**RMSE** ↓	**MAE** ↓	*R*^2^ ↑	**Pearson** ↑
Embedding dim = 64	0.195 ± 0.006	0.147 ± 0.005	0.858 ± 0.007	0.929 ± 0.006
Embedding dim = 128	0.186 ± 0.005	0.141 ± 0.004	0.871 ± 0.006	0.937 ± 0.005
Embedding dim = 256	0.190 ± 0.005	0.144 ± 0.004	0.865 ± 0.007	0.933 ± 0.005
Window length = 7	0.198 ± 0.007	0.149 ± 0.005	0.854 ± 0.008	0.927 ± 0.006
Window length = 14	0.186 ± 0.005	0.141 ± 0.004	0.871 ± 0.006	0.937 ± 0.005
Window length = 21	0.192 ± 0.006	0.145 ± 0.005	0.862 ± 0.007	0.931 ± 0.006
Penalty coefficient = 0.1	0.194 ± 0.006	0.147 ± 0.005	0.859 ± 0.007	0.930 ± 0.006
Penalty coefficient = 1.0	0.186 ± 0.005	0.141 ± 0.004	0.871 ± 0.006	0.937 ± 0.005
Penalty coefficient = 10.0	0.197 ± 0.007	0.149 ± 0.005	0.856 ± 0.008	0.928 ± 0.006
Missing input perturbation = 10%	0.204 ± 0.007	0.155 ± 0.005	0.844 ± 0.008	0.919 ± 0.007

All results are reported as mean ± standard deviation over 10 independent runs. ↑ indicates that higher is better, and ↓ indicates that lower is better.

#### 4.5.5 Empirical relevance in real public health settings

Beyond predictive comparison, the practical value of the present framework depends on whether its analytical structure corresponds to real public health policy settings. This relevance is supported by the empirical design of the study. Both OxCGRT and the CUSP dataset record actually implemented public health interventions together with region specific contextual variables and observed downstream responses. As a result, the model is trained on historically observed policy trajectories that emerged under real legal, administrative, and public health conditions, rather than on synthetic treatment assignments or abstract policy labels. This empirical grounding is important because public health decision making typically unfolds under conditions of uncertainty, institutional heterogeneity, delayed policy-associated outcome changes, and uneven regional capacity. The datasets used in this study capture variation in policy intensity, timing, and regional context across different jurisdictions and time periods. This makes them suitable for evaluating whether the proposed framework can identify policy response patterns that extend beyond a single region or a single governance setting. To further strengthen real world interpretability, an additional empirical assessment is conducted from the perspective of policy category level evaluation. Based on the policy taxonomy available in OxCGRT and CUSP, the observed interventions are grouped into four broad categories: containment and closure measures, health system measures, public information and monitoring measures, and socioeconomic support measures. For each category, the same temporal split, pre-processing pipeline, and evaluation protocol are retained, while the analysis focuses on samples in which the dominant intervention signal belongs to the corresponding category. This supplementary evaluation is intended to assess whether the proposed framework remains effective under intervention types that differ in administrative logic, implementation mechanism, and likely economic consequences. Containment and closure measures typically involve immediate restrictions on mobility and social interaction. Health system measures relate more directly to testing, tracing, vaccination, and care capacity. Public information and monitoring measures rely more heavily on communication and compliance behavior. Socioeconomic support measures aim to mitigate secondary harms associated with crisis response. These intervention categories are closely connected to real policy practice and therefore provide a more fine grained empirical perspective on model performance. The results are summarized in Table 13. Across all four categories, the proposed method maintains strong predictive performance, indicating that its empirical advantage is not limited to a single intervention domain. The performance gain is most pronounced under containment and closure measures, where delayed and non-linear economic responses are especially important, and remains stable under health system and socioeconomic support policies. These results strengthen the claim that the proposed framework has practical relevance for real public health environments in which policy instruments differ substantially in both design and downstream effects.

**Table 13:** Policy category level evaluation on representative intervention domains.

Policy category	RMSE ↓	MAE ↓	*R*^2^ ↑	Pearson correlation ↑
Containment and closure measures	0.221 ± 0.006	0.168 ± 0.005	0.836 ± 0.008	0.914 ± 0.007
Health system measures	0.217 ± 0.005	0.164 ± 0.004	0.840 ± 0.007	0.917 ± 0.006
Public information and monitoring measures	0.224 ± 0.006	0.170 ± 0.005	0.831 ± 0.008	0.910 ± 0.007
Socioeconomic support measures	0.219 ± 0.005	0.166 ± 0.004	0.838 ± 0.007	0.915 ± 0.006

All results are reported as mean ± standard deviation over 10 independent runs. Lower RMSE and MAE indicate better performance, while higher *R*^2^ and Pearson Correlation indicate better predictive quality.

#### 4.5.6 Case based validation

In addition to aggregate evaluation, a case based validation is introduced in order to examine whether the proposed framework remains informative in concrete public health policy episodes. The purpose of this analysis is not to replace large scale benchmarking, but to provide a more interpretable assessment of how the model behaves when confronted with historically observed intervention sequences that resemble actual policy decision settings. Representative policy episodes are constructed from the two datasets by selecting region time segments that exhibit clear changes in intervention intensity and sufficiently observable downstream economic response. Each case includes three elements: the policy configuration before adjustment, the observed legal or administrative intervention sequence, and the realized short horizon economic response in the subsequent period. The proposed framework is then used to generate predicted outcome changes under the observed intervention path, and its output is compared with the realized directional and relative magnitude pattern. This case based design allows a more concrete examination of whether the model captures practically meaningful response dynamics. In real public health governance, policymakers rarely compare isolated variables. Instead, they assess bundles of measures introduced under temporal pressure, legal constraints, and uncertain social response. A case oriented analysis is therefore useful for evaluating whether the model preserves interpretability when policy changes occur as structured sequences rather than as abstract inputs. Table 14 summarizes four representative cases. Across these cases, the proposed framework correctly captures the direction of the economic response and provides relatively close estimates of the magnitude of short horizon change. The model performs especially well in cases involving containment tightening and subsequent support adjustment, where the observed response depends on both direct restriction effects and compensatory policy mechanisms. These results do not amount to a full jurisdiction specific implementation study, but they provide an additional layer of empirical validation by demonstrating that the framework can produce interpretable outputs in policy episodes grounded in real public health intervention histories.

**Table 14:** Case based validation on representative public health policy episodes.

Case	Policy episode description	Dominant intervention type	Observed change	Predicted change	Interpretation
Case 1	Rapid tightening of mobility restriction and closure measures followed by a short observation window	Containment and closure	−0.31	−0.28	The model captures the immediate negative economic response associated with intensified restriction policy
Case 2	Expansion of testing and health system response under relatively stable closure policy	Health system measures	−0.08	−0.06	The framework reflects a milder short horizon economic effect under health system oriented intervention
Case 3	Introduction of public information measures with moderate administrative monitoring and limited restriction escalation	Information and monitoring	−0.05	−0.04	The predicted response remains small in magnitude and consistent with the observed directional pattern
Case 4	Restrictive measures combined with subsequent socioeconomic support adjustment	Mixed policy episode	−0.18	−0.15	The model captures the partially buffered economic decline associated with compensatory support intervention

Observed and predicted values denote normalized short horizon economic outcome change following the intervention episode.

### 4.6 Interpretability, sensitivity, and counterfactual analysis

**Figure 7:**
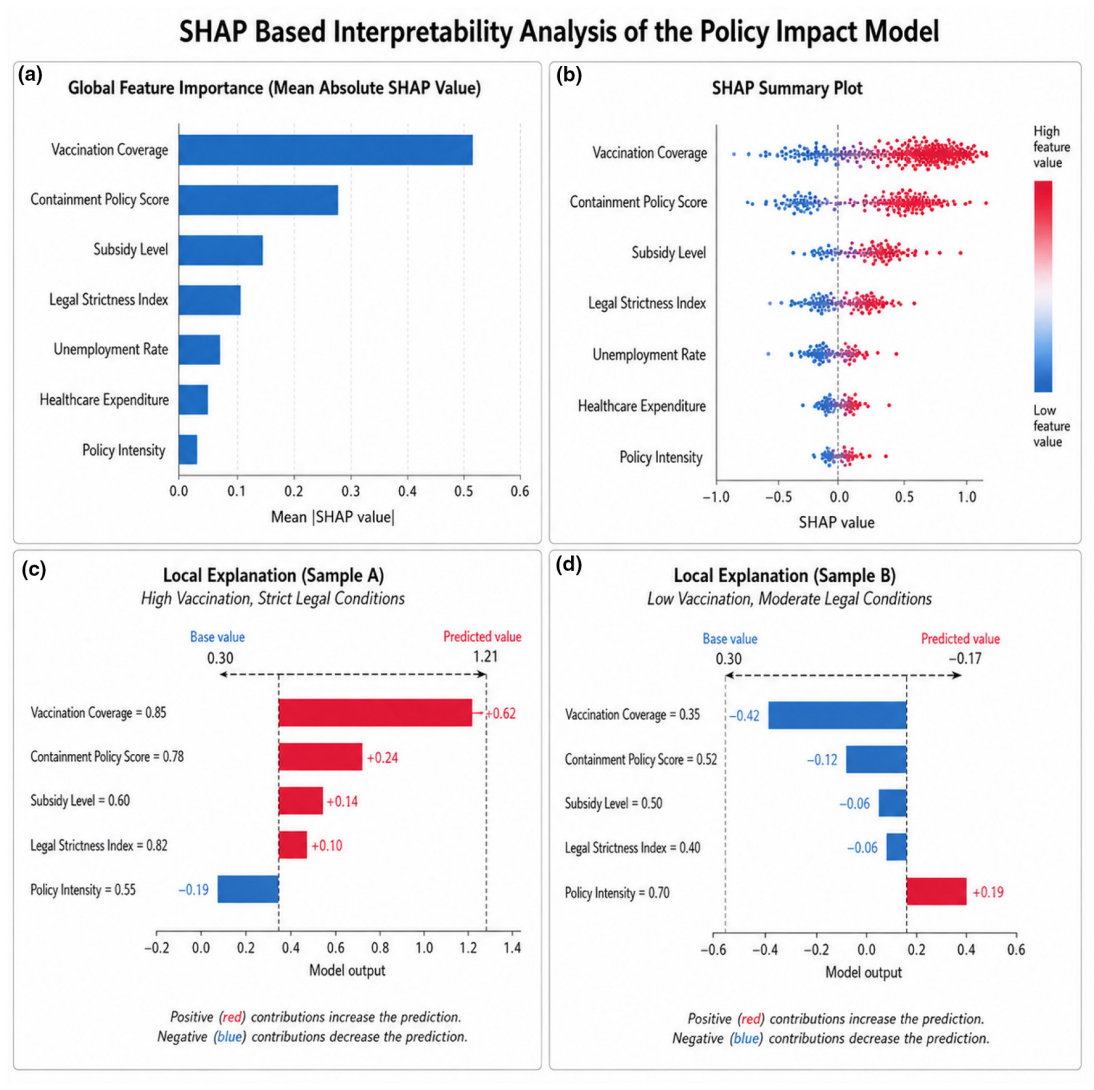
SHAP based interpretability analysis of the proposed policy-associated outcome prediction model. **(a)** Reports global feature importance measured by mean absolute SHAP values. **(b)** Presents the SHAP summary plot, where the horizontal axis represents the SHAP value and color indicates the feature value. **(c, d)** Show local explanations for two representative test samples, illustrating how key variables contribute positively or negatively to the predicted economic outcome change.

**Figure 8:**
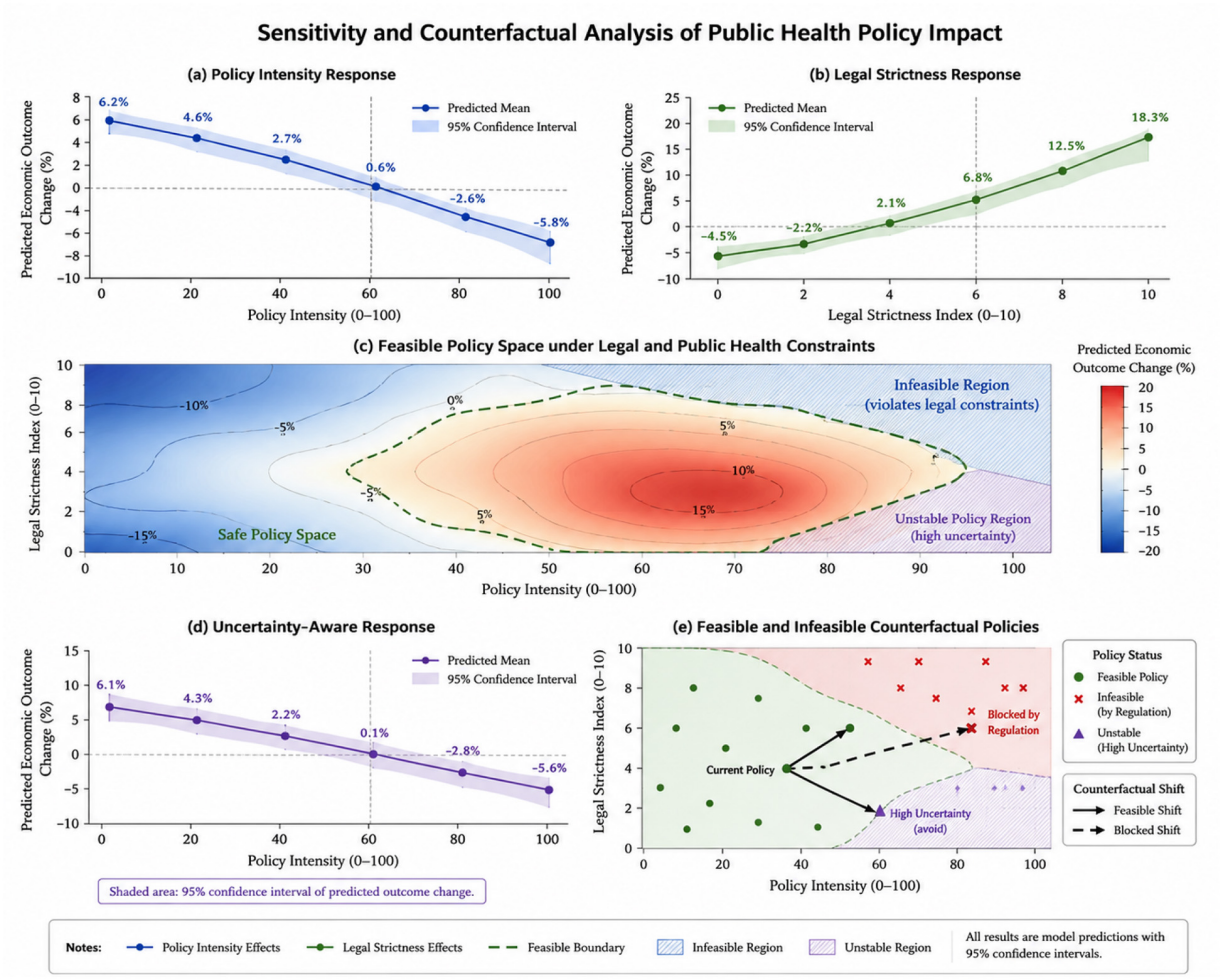
Sensitivity and counterfactual analysis of the proposed policy-associated outcome prediction model. **(a)** Shows the predicted economic response under different policy intensity levels. **(b)** Shows the response pattern under different legal strictness levels. **(c)** Visualizes the feasible policy space under legal and public health constraints, where infeasible and unstable policy regions are excluded. **(d)** Reports uncertainty aware predictions with confidence intervals. **(e)** Compares feasible and infeasible counterfactual policy scenarios and illustrates how regulatory constraints block policy choices outside the admissible region.

To improve the interpretability of the proposed policy-associated outcome prediction framework, this study adds SHAP based feature attribution, local explanation, sensitivity analysis, and counterfactual analysis. Since the proposed model contains non-linear temporal components and constrained policy representation, traditional regression coefficients alone are not sufficient to explain its prediction process. SHAP analysis is used as a model agnostic explanation tool to quantify the contribution of each input variable to the predicted economic outcome change. Figure 7 presents the SHAP based interpretability results. The global feature importance panel reports the mean absolute SHAP value of each input variable on the held out test set. A larger mean absolute SHAP value indicates a stronger contribution to the predicted economic response. The results show that vaccination coverage, containment policy score, subsidy level, legal strictness index, unemployment rate, health care expenditure, and policy intensity are among the major factors influencing the model prediction. This indicates that the proposed framework relies on both policy intervention variables and regional contextual variables when estimating economic outcome changes. The SHAP summary plot further explains the direction of influence. Positive SHAP values increase the predicted economic outcome change, while negative SHAP values decrease it. The color of each point represents the corresponding feature value, with higher feature values shown in red and lower feature values shown in blue. This visualization makes it possible to examine whether a higher value of a given variable tends to increase or decrease the predicted response. For example, higher vaccination coverage and stronger containment policy scores generally contribute positively in the illustrated results, while lower values of these variables tend to reduce the predicted economic response. The local explanation panels further show how individual predictions are formed. Sample A represents a case with high vaccination coverage and strict legal conditions. In this case, vaccination coverage, containment policy score, subsidy level, and legal strictness index provide positive contributions to the predicted output, while policy intensity contributes negatively. Sample B represents a case with lower vaccination coverage and moderate legal conditions. In this case, the lower vaccination coverage and weaker policy conditions reduce the predicted output, while increased policy intensity provides a partial positive contribution. These local explanations clarify how the same model produces different predictions under different policy and regional conditions. Figure 8 presents the sensitivity and counterfactual analysis. The policy intensity response panel shows how the predicted economic outcome changes when policy intensity varies from low to high levels. The legal strictness response panel shows how the predicted response changes under different levels of legal strictness. These response curves provide direct evidence on the direction and magnitude of key variable effects. The confidence intervals indicate prediction uncertainty and help distinguish stable response patterns from uncertain estimates. The feasible policy space panel further visualizes the interaction between policy intensity and legal strictness. The color surface represents the predicted economic outcome change, while the dashed boundary denotes the feasible policy space under legal and public health constraints. Regions outside the feasible boundary are marked as infeasible or unstable. This design shows that not all high outcome counterfactual policies are legally or practically admissible. The proposed framework does not only estimate predictive outcomes, but also evaluates whether a policy configuration lies within an acceptable decision space. The counterfactual policy panel compares feasible and infeasible policy alternatives. Feasible counterfactual shifts remain inside the legally admissible region, while blocked shifts are excluded by regulatory constraints. High uncertainty regions are also identified to avoid overinterpreting unstable predictions. This analysis supports the role of the legal constraint component in the proposed framework and clarifies how legal structures affect the set of policy choices available for evaluation. The added interpretability analysis reduces the black box nature of the proposed model. The SHAP results identify important variables and their contribution directions, while the sensitivity and counterfactual analyses show how changes in key policy and legal variables affect predicted economic outcomes. These results provide a more transparent explanation of how the proposed framework captures relationships among public health interventions, legal constraints, regional conditions, and economic consequences.

## 5 Discussion

### 5.1 Theoretical boundary of the present study

The framework proposed in this study evaluates public health policies through a formal structure that jointly considers economic variables and health outcomes. This formulation is introduced to support comparative policy analysis in a complex legal and socioeconomic environment. However, such a design should not be interpreted as offering a complete normative theory of public health governance. The utility based representation used in this study functions as an analytical instrument that renders policy consequences comparable within a tractable modeling setting. It does not claim that all public health values can be exhaustively reduced to aggregate welfare calculation. This theoretical boundary is especially important in view of the normative status of health. Within public health law and international human rights discourse, health is widely understood not only as a measurable social outcome but also as a fundamental right that gives rise to affirmative public obligations. Therefore, the present framework should not be read as suggesting that health protection is justified only when it produces favorable economic consequences, nor that the value of health can always be balanced against economic considerations on a purely quantitative basis. The model is designed to describe policy consequences, not to replace the independent normative force of health related legal commitments.

### 5.2 Positioning of the framework

The framework should be understood as a decision support tool rather than a self sufficient normative decision rule. Its primary purpose is to assist policymakers and researchers in identifying the likely consequences of alternative legal and policy arrangements, clarifying observable trade offs, and improving transparency in comparative evaluation. In this sense, the model contributes to empirical and institutional analysis of policy effectiveness, but it does not by itself determine what is legally justified or ethically required. Normative questions concerning constitutional commitments, minimum core obligations, equality guarantees, and the legal content of the right to health require external legal and ethical assessment that cannot be generated solely from model outputs. Even where the framework identifies a policy option with superior predicted aggregate performance, such a result must still be examined in light of rights based constraints, legality requirements, and the protection owed to vulnerable populations. The present framework is complementary to legal reasoning and public health ethics, rather than a substitute for them.

In practical terms, the CPO framework can support public decision making in several ways. It can be used for feasible policy scenario comparison. Health ministries, regional authorities, international organizations, or crisis preparedness institutions may compare predicted economic outcome changes under alternative public health interventions, such as targeted regional restrictions, broader mobility controls, vaccination related measures, workplace rules, or health system support policies. Because the model incorporates legal and regulatory constraints, these comparisons are conducted within a feasible policy space rather than among abstract policy options that may not be legally or administratively implementable. The framework can support economic risk estimation before policy adoption. By producing predicted economic outcome changes together with uncertainty information, CPO can help decision makers assess whether a proposed intervention is associated with relatively stable economic responses or with highly uncertain and potentially adverse outcomes. This information may support more cautious policy design, earlier preparation of fiscal support, targeted assistance for affected sectors, or adjusted implementation schedules before economic pressure becomes more pronounced. CPO can help identify regions where a public health measure may be associated with stronger economic responses. Regions differ in economic structure, health system capacity, legal conditions, administrative resources, digital readiness, and prior implementation histories. The same public health intervention may be associated with different predicted economic responses across jurisdictions. By comparing region time predictions, the framework can help identify areas that may require additional fiscal support, differentiated implementation strategies, closer public health monitoring, or stronger communication with affected communities. These uses remain within the predictive and associational scope of the study. The framework does not determine which policy should be adopted and does not provide causally identified policy effects.

### 5.3 Boundary of model applicability

The model is most appropriate for settings in which policymakers seek to compare alternative policy packages, implementation intensities, or regulatory combinations within an already specified legal environment. It is also suitable for scenario analysis in which economic and public health consequences can be represented through measurable indicators and where counterfactual comparison is necessary to support policy learning. Under such conditions, the framework can provide structured and interpretable guidance concerning the dynamic interaction between legal frameworks, economic effects, and public health outcomes. The framework is most useful in contexts with relatively rich and reliable data, clearly defined public health policies, stable legal and institutional environments, and sufficient administrative capacity for policy implementation and reporting. In such settings, policy variables, regulatory conditions, regional characteristics, and economic outcome indicators can be aligned into region time observations with reasonable consistency. By contrast, the results should be interpreted with caution in regions where data are incomplete, reporting systems are weak, legal frameworks change rapidly, or administrative capacity is limited. The model may also be less reliable when policy definitions are ambiguous, enforcement practices are highly informal, or economic responses are shaped by shocks that are not adequately represented in the available data. In such settings, model outputs should be treated as preliminary decision support rather than as a sufficient basis for policy choice. Additional legal analysis, local institutional knowledge, and expert judgment remain necessary.

At the same time, the applicability of the model remains limited in several important respects. The framework is not designed to determine the minimum core content of the right to health or to adjudicate cases in which basic health guarantees are at stake. It does not fully capture dignity based claims, non-quantifiable harms, or categorical legal prohibitions that may control policy choice independently of aggregate outcomes. In its current form, the model focuses primarily on aggregate policy performance and therefore cannot provide a complete account of distributive justice across social groups. Questions involving low income populations, geographically marginalized communities, persons with disabilities, and other vulnerable groups may require additional rights sensitive constraints and group specific equity analysis beyond the current model structure. These limitations define the appropriate scope of the present framework and indicate an important direction for future refinement.

### 5.4 Policy implementation as a relational and socially mediated process

The present framework models policy effectiveness through structured legal variables, economic indicators, and predicted public health outcomes. While this analytical design is useful for formal comparison, it does not imply that policy implementation should be understood as a purely technical stage that simply operationalizes prior decisions. In real public health governance, implementation is a relational and socially mediated process through which formal policy directives are interpreted, adapted, negotiated, and sometimes contested across different institutional and social settings. This perspective is important because the effectiveness of public policy depends not only on the formal content of a legal measure, but also on the interactions among the actors involved in its execution. Public authorities, local administrative bodies, healthcare institutions, frontline professionals, community organizations, and affected populations all participate in shaping how a policy is translated into practice. Differences in institutional trust, administrative capacity, professional discretion, local coordination, and public compliance may substantially alter policy outcomes even when the formal legal rule remains unchanged. From this standpoint, implementation cannot be reduced to a neutral transmission mechanism between policy design and policy outcome. It is better understood as a socially embedded process in which legal norms encounter institutional structures and lived social conditions. Public health interventions are often realized through multilevel systems of governance, where central directives depend on local enforcement capacity, interagency cooperation, and community acceptance. As a result, the same policy design may generate different effects across jurisdictions or populations because implementation is mediated by distinct relational environments. This conceptual clarification also helps define the interpretive boundary of the present model. The framework is designed to analyze policy consequences on the basis of structured and observable variables, but it does not fully capture the social processes through which implementation unfolds in practice. In particular, informal negotiation, institutional friction, professional judgment, and community response remain only partially visible within the current modeling structure. The model should therefore be understood as capturing important outcome related dimensions of policy effectiveness without claiming to exhaust the relational mechanisms that shape implementation in real world settings. Recognizing the relational character of implementation suggests an important direction for future research. Further extensions may incorporate governance quality indicators, institutional capacity measures, proxies for public trust and compliance, and case based evidence on local implementation dynamics. Such developments would allow the analytical framework to better connect formal policy design with the socially mediated processes through which public health interventions acquire their practical effectiveness.

The empirical findings of this study are consistent with and extend prior research on legal frameworks, regulatory compliance, administrative capacity, health equity, and predictive policy analysis. Existing public health governance literature emphasizes that policy outcomes depend on institutional capacity, enforcement conditions, resource allocation, and social acceptability. The present results support this view by showing that predictive performance improves when legal feasibility, regional heterogeneity, and uncertainty are incorporated into the estimation framework. The findings also extend studies on regulatory compliance costs by treating economic response not only as an external consequence of policy implementation, but also as a central prediction target that affects the sustainability of public health interventions. The results also speak to the literature on algorithmic and predictive models for public policy. General machine learning approaches can capture non-linear patterns in policy data, but they often treat policy variables as unconstrained inputs and do not explicitly represent legal feasibility or institutional implementation conditions. By contrast, the present framework embeds legal constraints, actor sensitive policy interactions, and probabilistic forecasting into the model structure. In this sense, the findings expand existing predictive policy research by showing that performance gains can arise not only from greater algorithmic capacity, but also from aligning model design with the legal and institutional characteristics of public health governance. If differences from some conventional econometric studies arise, they may reflect the different empirical goal of this study: supervised prediction of policy associated economic outcome changes rather than causally identified estimation of policy effects.

### 5.5 Ethical and accountability considerations

The use of predictive models in public health governance raises important ethical and accountability concerns. Model outputs depend on the quality, completeness, and representativeness of the underlying data. If policy records, economic indicators, or public health measurements are biased or unevenly reported across regions, the predictions may reproduce those biases. Regions with weaker data infrastructure may appear less predictable, not necessarily because policy responses are inherently more unstable, but because the available information is incomplete or inconsistent. Algorithmic opacity may limit public accountability. Even when a model produces accurate predictions, decision makers and affected communities need to understand why a scenario is classified as risky, feasible, or uncertain. If model outputs are treated as authoritative without sufficient explanation, they may weaken democratic oversight and reduce the visibility of legal and ethical reasoning. For this reason, CPO should be used together with interpretability tools, legal review, public health expertise, and institutional deliberation. Predictive models may reproduce or amplify existing regional inequalities. Areas with lower administrative capacity, weaker digital readiness, or poorer health system resources may be assigned higher predicted economic risk. Such results can be useful for identifying vulnerability, but they may also lead to stigmatization or reduced policy ambition if interpreted without equity safeguards. High predicted risk should be used to trigger additional support and careful implementation planning, rather than to justify exclusion from public health protection. Responsibility for public decisions cannot be delegated to automated recommendations. The framework provides structured evidence on predicted economic responses, legal feasibility, and uncertainty, but elected officials, public health authorities, legal institutions, and administrative bodies remain responsible for policy choices. Algorithmic outputs should inform deliberation, not replace accountable decision making. This distinction is particularly important in public health emergencies, where rapid decisions may have substantial consequences for rights, livelihoods, and access to essential services.

### 5.6 Uncertainty as a component of public health governance

Uncertainty in this study should not be understood merely as technical noise or model imperfection. It is a substantive component of public health governance. Public health policies are adopted under incomplete information, changing epidemiological conditions, heterogeneous regional capacities, and evolving social responses. As a result, uncertainty is present not only in model estimation, but also in the policy environment itself. The probabilistic forecasting component of CPO is therefore important because it communicates the degree of uncertainty surrounding predicted economic outcome changes. A prediction with low uncertainty may indicate that similar policy and regional conditions in the observed data are associated with relatively stable economic responses. A prediction with high uncertainty may indicate that the relevant conditions are heterogeneous, weakly represented in the data, or subject to unstable implementation dynamics. For decision makers, this distinction is important. It can help avoid overly confident conclusions, encourage additional monitoring, support contingency planning, and justify complementary measures when the predicted response is highly uncertain. In this sense, uncertainty aware prediction can improve the prudence of public health decision making. Rather than presenting a single deterministic forecast, the framework allows policymakers to distinguish between scenarios that are comparatively stable and scenarios that require caution. This approach is aligned with the broader governance need to make decisions under uncertainty while remaining transparent about the limits of available evidence.

## 6 Conclusions and future work

This study developed a predictive computational framework for estimating economic outcome changes associated with legal frameworks and public health policy conditions. Rather than treating the problem as causal effect identification, the study formulated the empirical task as supervised temporal regression based on observational region–time panel data. The proposed framework integrates three core components: Preliminaries, Counterfactual Policy Optimizer (CPO), and Counterfactual Pacing with Probabilistic Policy Outcome Refinement. Through the Preliminaries, we established a formal representation of legal and regulatory variables, economic factors, public health indicators, and feasible policy constraints. The CPO incorporated the Manifold Constraint Regularizer, Agent-driven Event Planner, and Probabilistic Outcome Forecaster to model non-linear temporal associations between observed policy conditions and subsequent economic outcome changes. The Counterfactual Pacing and Probabilistic Policy Outcome Refinement strategy further supported model-based scenario comparison and uncertainty-aware prediction refinement.

Experimental results on OxCGRT and CUSP suggest that the proposed framework achieves improved predictive accuracy and trend consistency compared with traditional econometric, machine learning, and temporal deep learning baselines under the same observational policy outcome estimation setting. These results indicate that incorporating legal feasibility constraints, agent-driven interaction modeling, and probabilistic forecasting can provide a structured computational tool for analyzing policy-associated economic responses. The contribution of this study is not limited to improving predictive accuracy. More importantly, it provides a public health policy support framework that jointly considers legal constraints, economic risks, institutional feasibility, and predictive uncertainty. This perspective is relevant to public health because policy evaluation often requires assessing not only whether an intervention is associated with favorable health or economic outcomes, but also whether it can be sustained under available resources, implemented within legal and administrative boundaries, and interpreted responsibly under uncertainty. In this sense, the study contributes to public health research by linking computational prediction with health governance, legal feasibility, and economic risk awareness. The findings should be interpreted as evidence of predictive associations and temporal correspondence, rather than as causal estimates of the effects of legal frameworks or public health policies. The model-based scenario comparisons reported in this study are intended to support predictive policy analysis within the observed data distribution and feasible legal-policy space, not to establish causally identified counterfactual outcomes.

Despite these promising predictive results, there are several notable limitations in this study. The framework relies heavily on the quality, consistency, and availability of input data, such as policy records, legal and regulatory indicators, economic variables, and public health metrics. In scenarios where data are sparse, delayed, inconsistent, or affected by heterogeneous reporting practices, the accuracy and reliability of the model's predictions may be compromised. Future research should focus on developing more robust methods for handling incomplete, noisy, or irregularly sampled policy panel data. While the CPO provides a structured tool for uncertainty-aware prediction and model-based scenario comparison, its computational complexity can pose challenges for real-time policy analysis and decision support. Optimizing the framework for scalability and efficiency will be important for its practical use in dynamic and fast-evolving public health contexts. A further limitation is that the present framework does not provide causal identification. The analysis is conducted on observational panel data, and the proposed model does not rely on a structural causal model, randomized intervention, instrumental variable, regression discontinuity design, synthetic control construction, or other exogenous source of variation required for causal inference. Therefore, the estimated relationships may still be affected by unobserved confounding, policy endogeneity, simultaneous regional shocks, and measurement heterogeneity. Future work could integrate the proposed predictive architecture with explicit causal identification strategies, such as difference-in-differences with stronger design diagnostics, instrumental variables, regression discontinuity designs, synthetic control methods, or structural causal models. Such extensions would make it possible to examine whether the predictive associations captured by the current framework can be connected to causally interpretable policy effects under stronger identification assumptions.

Several additional directions for future research are worth emphasizing. The framework could be extended beyond the COVID-19 policy context to other public health domains, such as vaccination policy, chronic disease prevention, occupational health regulation, environmental health interventions, and preparedness for future health emergencies. These settings also involve interactions among legal authority, administrative capacity, economic burden, and public health outcomes, making them suitable for legally constrained and uncertainty-aware policy prediction. Future work should incorporate more explicit equity and vulnerability indicators. The present framework mainly models aggregate economic outcome changes, but public health evaluation requires attention to how policy-associated responses are distributed across population groups. Future extensions could include variables related to social inequality, access to healthcare services, regional deprivation, economic vulnerability, disability status, age structure, occupational exposure, and the distribution of policy outcomes across disadvantaged groups. Such development would make the framework more responsive to the fact that aggregate averages may conceal unequal burdens and benefits. More refined legal and institutional indicators could also be incorporated, including measures of enforcement capacity, administrative accountability, judicial review, rights protection, emergency powers, and procedural safeguards. These additions would improve the ability of the framework to represent the legal and institutional conditions under which public health policies are implemented. Institutional validation should be conducted in collaboration with experts in public policy, health law, public health governance, and health system management. Such validation could assess whether the predictions, uncertainty estimates, and feasibility indicators generated by the model are interpretable, useful, and acceptable to decision makers. It could also help determine how the framework may be integrated into real-world decision support systems without replacing political, legal, or ethical deliberation. Addressing these limitations will further enhance the transparency, reliability, and practical value of computational approaches for integrating legal, economic, and public health considerations in policy analysis.

## Data Availability

The original contributions presented in the study are included in the article/supplementary material, further inquiries can be directed to the corresponding author.

## References

[1] Greer SL Fonseca EM Raj M Willison CE. Institutions and the politics of agency in COVID-19 response: federalism, executive power, and public health policy in Brazil, India, and the US. J Soc Policy. (2024) 53:792–810. doi: 10.1017/S0047279422000642

[2] Burris S Wagenaar AC Swanson J Ibrahim JK Wood J Mello MM. Making the case for laws that improve health: a framework for public health law research. Milbank Q. (2010) 88:169–210. doi: 10.1111/j.1468-0009.2010.00595.x 20579282 PMC2980343

[3] Gostin LO Magnusson RS Krech R Patterson DW Solomon SA Walton D . Advancing the right to health—the vital role of law. Am J Public Health. (2017) 107:1755. doi: 10.2105/AJPH.2017.304077 29019787 PMC5637685

[4] Braveman P. Defining health equity. J Natl Med Assoc. (2022) 114:593–600. doi: 10.1016/j.jnma.2022.08.004 36167751

[5] Braveman P Kumanyika S Fielding J LaVeist T Borrell LN Manderscheid R . Health disparities and health equity: the issue is justice. Am J Public Health. (2011) 101:S149–55. doi: 10.2105/AJPH.2010.300062 21551385 PMC3222512

[6] Monroe P Campbell JA Harris M Egede LE. Racial/ethnic differences in social determinants of health and health outcomes among adolescents and youth ages 10-24 years old: a scoping review. BMC Public Health. (2023) 23:410. doi: 10.1186/s12889-023-15274-x 36855084 PMC9976510

[7] Marmot M Friel S Bell R Houweling TAJ Taylor S. Closing the gap in a generation: health equity through action on the social determinants of health. Lancet. (2008) 372:1661–9. doi: 10.1016/S0140-6736(08)61690-6 18994664

[8] Jit M Cook AR. Informing public health policies with models for disease burden, impact evaluation, and economic evaluation. Annu Rev Public Health. (2024) 45:025149. doi: 10.1146/annurev-publhealth-060222-025149 37871140

[9] Williams DR Mohammed SA. Racism and health I: pathways and scientific evidence. Am Behav Sci. (2013) 57:1152–73. doi: 10.1177/0002764213487340 24347666 PMC3863357

[10] Baicker K Taubman SL Allen HL Bernstein M Gruber JH Newhouse JP . The Oregon experiment: effects of Medicaid on clinical outcomes. N Engl J Med. (2013) 368:1713–22. doi: 10.1056/NEJMsa1212321 23635051 PMC3701298

[11] Luyten E Tubeuf S. Equity in healthcare financing: a review of evidence. Health Policy. (2025) 152:105218. doi: 10.1016/j.healthpol.2024.105218 39740647

[12] Courtemanche C Marton J Ukert B Yelowitz A Zapata D. Early impacts of the affordable care act on health insurance coverage in Medicaid expansion and non expansion states. J Policy Anal Manag. (2017) 36:178–210. doi: 10.1002/pam.21961 27992151

[13] Sommers BD Blendon RJ Orav EJ Epstein AM. Changes in utilization and health among low income adults after medicaid expansion or expanded private insurance. JAMA Intern Med. (2016) 176:1501–9. doi: 10.1001/jamainternmed.2016.4419 27532694

[14] Sommers BD Maylone B Blendon RJ Orav EJ Epstein AM. Three Year Impacts of the affordable care act: improved medical care and health among low income adults. Health Aff . (2017) 36:1119–28. doi: 10.1377/hlthaff.2017.0293 28515140

[15] Antonisse L Garfield R Rudowitz R Artiga S. The Effects of Medicaid Expansion Under the ACA: Updated Findings from a Literature Review (2018). p. 28. Available online at: https://nationaldisabilitynavigator.org/wp-content/uploads/news-items/KFF_Effects-of-Medicaid-Expansion-Lit-Review_Feb-2017-chart.pdf

[16] Kominski GF Nonzee NJ Sorensen A. The affordable care act's impacts on access to insurance and health care for low income populations. Annu Rev Public Health. (2017) 38:489–505. doi: 10.1146/annurev-publhealth-031816-044555 27992730 PMC5886019

[17] Cheng C Barceló J Hartnett AS Kubinec R Messerschmidt L. COVID-19 government response event dataset CoronaNet v10. Nat Hum Behav. (2020) 4:756–68. doi: 10.1038/s41562-020-0909-7 32576982

[18] Haug N Geyrhofer L Londei A Dervic E Desvars Larrive A Loreto V . Ranking the effectiveness of worldwide COVID-19 government interventions. Nat Hum Behav. (2020) 4:1303–12. doi: 10.1038/s41562-020-01009-0 33199859

[19] Brauner JM Mindermann S Sharma M Johnston D Salvatier J Gavenĉiak T . Inferring the effectiveness of government interventions against COVID-19. Science. (2021) 371:eabd9338. doi: 10.1126/science.abd9338 33323424 PMC7877495

[20] Flaxman S Mishra S Gandy A Unwin HJT Mellan TA Coupland H . Estimating the effects of non pharmaceutical interventions on COVID-19 in Europe. Nature. (2020) 584:257–61. doi: 10.1038/s41586-020-2405-7 32512579

[21] Mendez Brito A El Bcheraoui C Pozo Martin F. Systematic review of empirical studies comparing the effectiveness of non pharmaceutical interventions against COVID-19. J Infect. (2021) 83:281–93. doi: 10.1016/j.jinf.2021.06.018 34161818 PMC8214911

[22] Bendavid E Oh C Bhattacharya J Ioannidis JPA. Assessing mandatory stay at home and business closure effects on the spread of COVID-19. Eur J Clin Invest. (2021) 51:e13484. doi: 10.1111/eci.13484 33400268 PMC7883103

[23] Abadie A. Using Synthetic controls: feasibility, data requirements, and methodological aspects. J Econ Lit. (2021) 59:391–425. doi: 10.1257/jel.20191450

[24] Levy K Chasalow KE Riley S. Algorithms and decision-making in the public sector. Annu Rev Law Soc Sci. (2021) 17:309–34. doi: 10.1146/annurev-lawsocsci-041221-023808

[25] Criado JI Sandoval-Almazán R Gil-Garcia JR. Artificial intelligence and public administration: understanding actors, governance, and policy from micro, meso, and macro perspectives. Public Policy Administr. (2025) 40:173–84. doi: 10.1177/09520767241272921

[26] Vatamanu AF Tofan M. Integrating artificial intelligence into public administration: challenges and vulnerabilities. Administr Sci. (2025) 15:149. doi: 10.3390/admsci15040149

[27] Alshahrani A Griva A Dennehy D Mäntymäki M. Artificial intelligence and decision-making in government functions: opportunities, challenges and future research. Transform Govern: People Process Policy. (2024) 18:678–98. doi: 10.1108/TG-06-2024-0131

[28] Joshi D. Algorithmic Accountability for the Public Sector: Learning from the First Wave of Policy Implementation (2021). Available online at: https://discovery.ucl.ac.uk/id/eprint/10193737/1/algorithmic-accountability-public-sector.pdf.

[29] Zlati ML Fortea C Antohi VM Cristache N Dinca MS Balsalobre-Lorente D. Refreshing the design of a regional economic growth model in the context of the new digital decade. Technol Econ Dev Econ. (2026) 32:336–73. doi: 10.3846/tede.2025.24534

[30] Zlati ML Beldiman CM Vasiliev I Antohi VM. Fortea C. Regional disparities in the transition to industry 50: an algorithmic approach to digitization. Econ Change Restruct. (2026) 59:67. doi: 10.1007/s10644-026-09988-2

[31] Hale T Angrist N Goldszmidt R Kira B Petherick A Phillips T . A global panel database of pandemic policies (Oxford COVID-19 Government Response Tracker). Nat Hum Behav. (2021) 5:529–38. doi: 10.1038/s41562-021-01079-8 33686204

[32] Skinner A Flannery K Nocka K Bor J Dean LT Jay J . A database of US state policies to mitigate COVID-19 and its economic consequences. BMC Public Health. (2022) 22:1124. doi: 10.1186/s12889-022-13487-0 35659285 PMC9166228

[33] Saeed S Moodie EE Strumpf EC Klein MB. Evaluating the impact of health policies: using a difference-in-differences approach. Int J Public Health. (2019) 64:637–42. doi: 10.1007/s00038-018-1195-2 30607473

[34] Folorunsho O Fagbuagun A Nwankwo O Akinpelu S. Prediction of the effectiveness of government measures towards COVID-19 using multiple linear regression analysis. Univ Ibadan J Sci Logics ICT Res. (2021) 7:10–8. Available online at: https://journals.ui.edu.ng/index.php/uijslictr/article/view/723

[35] Wang H Song Y Bi H. Optimizing public health management with predictive analytics: leveraging the power of random forest. Front Big Data. (2025) 8:1574683. doi: 10.3389/fdata.2025.1574683 40708674 PMC12286995

[36] Verma I Prasad SK. Advancing public health initiatives: a comprehensive analysis of infant mortality predictors in India using the robust XGBoost algorithm. Int J Inform Technol. (2025) 17:5637–44. doi: 10.1007/s41870-025-02467-3

[37] Absar N Uddin N Khandaker MU Ullah H. The efficacy of deep learning based LSTM model in forecasting the outbreak of contagious diseases. Infect Dis Modell. (2022) 7:170–83. doi: 10.1016/j.idm.2021.12.005 34977438 PMC8712463

[38] Caldas FM Soares C. A temporal fusion transformer for long-term explainable prediction of emergency department overcrowding. In: Joint European Conference on Machine Learning and Knowledge Discovery in Databases. Cham: Springer (2022). p. 71–88. doi: 10.1007/978-3-031-23618-1_5

